# Efficacy and safety of Atractylodes macrocephala-containing traditional Chinese medicine combined with neoadjuvant chemotherapy in the treatment of advanced gastric cancer: a systematic evaluation and meta-analysis

**DOI:** 10.3389/fonc.2024.1431381

**Published:** 2024-10-16

**Authors:** Xiaotao Niu, Haoqing Gu, Jingzhan Li, Jiaqian Zuo, Wenqin Ren, Yujie Huang, Xinyan Shu, Chao Jiang, Peng Shu

**Affiliations:** ^1^ Affiliated Hospital of Nanjing University of Traditional Chinese Medicine, Nanjing, Jiangsu, China; ^2^ School of No. 1 Clinical Medical, Nanjing University of Chinese Medicine, Nanjing, Jiangsu, China

**Keywords:** *Atractylodes macrocephala*, neoadjuvant chemotherapy, progressive gastric cancer, traditional Chinese medicine, meta-analysis

## Abstract

**Background:**

In China, Atractylodes-containing Chinese medicines are widely used as adjuvant therapy to neoadjuvant chemotherapy (NAC) in individuals diagnosed with advanced gastric cancer (AGC). Nevertheless, the findings concerning its effectiveness are still restricted. The aim from this research was to examine the efficiency and security Atractylodes macrocephala-containing traditional Chinese medicine together with NAC in the management of AGC.

**Methods:**

Literature was systematically searched across 8 electronic databases until September 20, 2023. Two researchers conducted a thorough review of the selected studies. The primary outcome measures included the objective response rate (ORR), disease control rate (DCR), quality of life (QOL), adverse drug reactions (ADRs), and levels of peripheral blood lymphocytes. The relevant effect estimates are as follows as risk ratios (RR) or mean differences (MD) with corresponding 95% confidence intervals (CI). Credibility of information was evaluated using the GRADE analyzer.

**Results:**

The results showed that solely on the basis of the accessible literature examined in NAC patients, individuals who received the therapeutic regimen containing Atractylodis Macrocephalae Chinese herbal preparations demonstrated a superior overall response rate (Relative Risk: 1.41, 95% confidence interval: 1.27-1.57, P < 0.001); DCR (RR: 1.20, 95% confidence interval: 1.13-1.27, P < 0.001), as compared to QOL (RR: 1.43, 95% confidence interval: 1.30-1.57, P < 0.001, MD: 8.47, 95% confidence interval: 7.16 - 9.77, P < 0.001); the proportions of CD3^+^ T-cells, CD4^+^ T-cells, CD8^+^ T-cells, CD4^+^CD8^+^ T-cells were increased; and the incidence of adverse reactions was decreased. Subgroup analyses showed that oral administration of all the traditional Chinese medicines containing Atractylodes macrocephala could improve tumor efficacy. Regardless of the duration of therapy of ≥8 weeks or <8 weeks, Atractylodes macrocephala-containing traditional Chinese medicine increased the tumor response in AGC patients. Combination of Atractylodes macrocephala-containing TCM with neoadjuvant chemotherapy increased ORR and DCR; when used in conjunction with cisplatin, only ORR was increased.

**Conclusion:**

The combination of Atractylodes macrocephala-containing herbs with NAC in the treatment of AGC improves efficacy, improves prognosis, and reduces adverse effects. Nevertheless, additional high-quality randomized trials are required.

**Systematic review registration:**

https://www.crd.york.ac.uk/PROSPERO/, identifier CRD42023461079.

## 1 Introduction

Gastric cancer, among the most prevalent tumors in humans, continues to be the third primary reason for cancer-related deaths globally, it ranks 5th with regard to prevalence and 4th with regard to mortality (1, 2). The key symptoms of gastric cancer include weight loss, indigestion, nausea, vomiting, loss of appetite, and difficulty swallowing (3). Nonetheless, early-stage gastric cancer often presents with subtle clinical manifestations, leading to many patients being diagnosed at an advanced stage (4). While surgical intervention continues to be the main approach option for advanced gastric cancer, research findings indicate that over 50% of patients experience recurrence within five years post-surgery (5).

According to the Cancer Guidelines of the Chinese Clinical Society (version 2023) (6), neoadjuvant chemotherapy is generally used for advanced gastric cancer, including the combination of fluorouracil (capecitabine [CAP], tegor), platinum (cisplatin [DDP],oxaliplatin [OXA]), taxoid drugs, etc. Neoadjuvant chemotherapy can reduce the mortality of advanced gastric cancer and esophageal gastric cancer, reduce the total recurrence rate of esophageal gastric cancer, and improve the pathologic complete response (pathCR) rate of patients with advanced gastric cancer (7, 8). Perioperative chemotherapy of Epirubicin, cisplatin and fluorouracil can reduce the size and stage of tumors in patients with operable gastric or lower esophageal adenocarcinomas, the patients’ progression-free survival and overall survival were significantly improved (9). However, neoadjuvant chemotherapy may also cause certain damage to the body function of patients and produce drug resistance (10), which limits the clinical efficacy, thus prompting researchers and clinicians to pay more attention to the study of alternative and complementary therapies.

Atractylodes macrocephala, the root of the chrysanthemum plant, has been considered one of the main tonic herbs in traditional Chinese medicine for more than 2,000 years and is widely used in China to treat malignant tumors. By conducting network pharmacology studies and *in vitro* and *in vivo* experiments on Atractylodes macrocephala, it is demonstrated that palmitic acid, atractylenolide I, beta-caryophyllene, D-Serine, alpha-humulene, atractylenolide III and other Atractylenolide extracts can be used as active substances to specifically target genes associated with gastric cancer (11). A basic experimental study (12), showed that Chinese traditional medicine compound containing Atractylodes macrocephala combined with oxaliplatin could affect the apoptosis of gastric cancer cells in tumor-bearing mice through the PI3K/Akt/caspase-9 signaling pathway.

In recent decades, a large number of trials containing Atractylodes macrocephala Chinese medicine combined with NAC have been published in the treatment of AGC. The purpose of this study was to conduct a systematic analysis of these findings to evaluate the safety and efficacy of Atractylodes macrocephala Chinese medicine combined with NAC in the treatment of AGC to provide a more solid evidence base for further clinical studies, and further explore the application potential of Atractylodes macrocephala in the treatment of gastric cancer.

## 2 Methods

This research has been carried out adhering to the Preferred Reporting Items for Systematic Reviews and Meta-Analyses (PRISMA) guidelines. The project is incorporated with PROSPERO under registration number CRD42023461079.

### 2.1 Search strategy

We conducted a systematic search in Web of Science, PubMed, EMBASE, Cochrane Central Register of Controlled Trials (Central), clinicaltrials.gov, China National Knowledge Infrastructure (CNKI), China Academic Journals (CQVIP), SINOMED and Wanfang databases were systematically searched to find articles published up to September 20, 2023: (gastric* or stomach* or digestive* or gastrointestinal*]. A systematic search of the China Academic Journals (CD-ROM Version) (CQVIP) and Wanfang databases was conducted to find articles published until September 20, 2023: (gastric* or stomach* or digestive* or gastrointestinal*] and [carcinoma* or cancer* or neoplasm* or tumour* or tumor* or tumor* or growth* or adenocarcinoma* or malignant*]) as well as (Atractylodes macrocephalus or Cangzhu). All queries used MeSH terms and non-restrictive vocabulary (detailed search strategies are provided in the **Supplementary Material**). Only Chinese and English were included.

### 2.2 Inclusion and exclusion criteria

Inclusion criteria: (1) All research included in the study were randomized controlled trials (RCT) or quasi-RCTs. (2) Patients diagnosed with TNM stage II-IV AGC using histopathological and cytological criteria. (3) Patients in the experimental group were given a combination of white-herb-based traditional Chinese medicine and neoadjuvant chemotherapy. Individuals in the control group were given neoadjuvant chemotherapy treatment alone. (4) The outcomes assessed in this study included tumor response, quality of life, adverse events, and peripheral blood lymphocyte levels, with each study reporting at least one of these outcomes. (5) In cases where multiple publications of the same study were available, we chose the dataset that provided the most exhaustive information and had the longest follow-up time.(6)The papers included in this study were published before September 23, 2023.

Exclusion criteria: (1) Exclusion of individuals with serious infections, other malignancies, and severe medical conditions; (2) Avoidance of inconsistent prescriptions of traditional Chinese medicines based on Atractylodes macrocephala;(3) Studies from which it was not possible to extract data were excluded; (4) Baseline data were not comparable between the two groups of patients.

### 2.3 Study selection and data extraction

Two reviewers independently selected studies based on the specified inclusion and exclusion criteria. Data extraction was also carried out independently by two reviewers. Whatever discrepancies there are will be resolved through discussion the third reviewer. The data collected shall include the first author and publication year, sample size, gender distribution, age range, study group details, dosage regimen, treatment duration, outcomes, and assessment criteria.

### 2.4 Risk of bias assessment

Two reviewers independently assessed the quality of the included studies utilizing the Cochrane Collaboration’s Tool for Randomized Controlled Trials.

These studies will be categorized as low, unclear, or high risk of bias. Assessment criteria included random sequence generation (selection bias), allocation concealment (selection bias), blinding of participants and staff (performance bias), incomplete outcome data (attrition bias), selective reporting (reporting bias), and other potential biases. The results of these assessments have all been summarized and analyzed through Review Manager 5.3.

### 2.5 Outcome definition

Tumor response is the main prognostic indicator. Tumor response includes objective remission rate (ORR) and disease control rate (DCR) based on World Health Organization (WHO) criteria (13) and solid tumor criteria (RECIST) (14). Complete remission (CR), partial remission (PR), stable disease (SD) and progression (PD) were used as indicators.

The remaining observations were quality of life (QOL), adverse drug reactions (ADRs) and peripheral blood lymphocyte levels.

Adverse reactions were obtained by measuring hematological toxicity (leukocyte abnormalities, thrombocytopenia, anemia), gastrointestinal toxicity (nausea, vomiting, and diarrhea), hepatic and renal dysfunction, neurotoxicity, alopecia, and stomatitis, and were assessed according to World Health Organization (WHO) criteria (13) or the National Institutes of Health Common Terminology Criteria for Adverse Events (CTCAE) (15). Peripheral blood lymphocyte levels were assessed by measuring T lymphocyte subsets such as CD3^+^ T cells, CD4^+^ T cells, and CD8^+^ T cells, as well as the CD4^+^/CD8^+^ T cell ratio.

### 2.6 Data analysis

Review Manager 5.3 and Stata 17.0 software were utilized for data analysis in this research. Risk ratios (RR) and 95% confidence intervals (CI) were employed to evaluate binary variables, while mean difference (MD) with 95% CI was used for assessment of continuous variables.

Statistical heterogeneity was assessed using the I^2^ statistic and chi-square test. Total risk ratios (RR), mean differences (MD), and their respective 95% confidence intervals were estimated through random-effects modeling. The significance of the results was determined by P values; statistical significance was defined when P < 0.05. Funnel plots and Egger’s tests were employed to examine possible publication bias when I^2^ ≥ 50%. Subgroup analyses were conducted based on first treatment, NAC regimen, and treatment duration to explore clinical heterogeneity and its effect on tumor response.

### 2.7 Evidence quality assessment

Independent review by two reviewers evaluated the level of evidence for each result utilizing the GRADE method (16). Any discrepancies were discussed and resolved with the involvement of a third reviewer. The quality of evidence is categorized as high, moderate, low, and very low. The evidence was only reduced by a single level if most trials exhibited unclear or elevated risk of bias but had robust results; a two-level downgrade occurred if the results lacked robustness. Other factors for downgrading included inconsistency (presence of statistical heterogeneity and poor robustness of the results of the sensitivity analyses), indirectness (the study’s participants, interventions, outcomes, or comparisons did not align with the study objectives), imprecision (sample size fewer than 300 cases for each outcome), and reporting bias (publication bias). Apart from the risk of bias, the evidence was downgraded by one level.

## 3 Results

### 3.1 Search results

**Figure 1:**
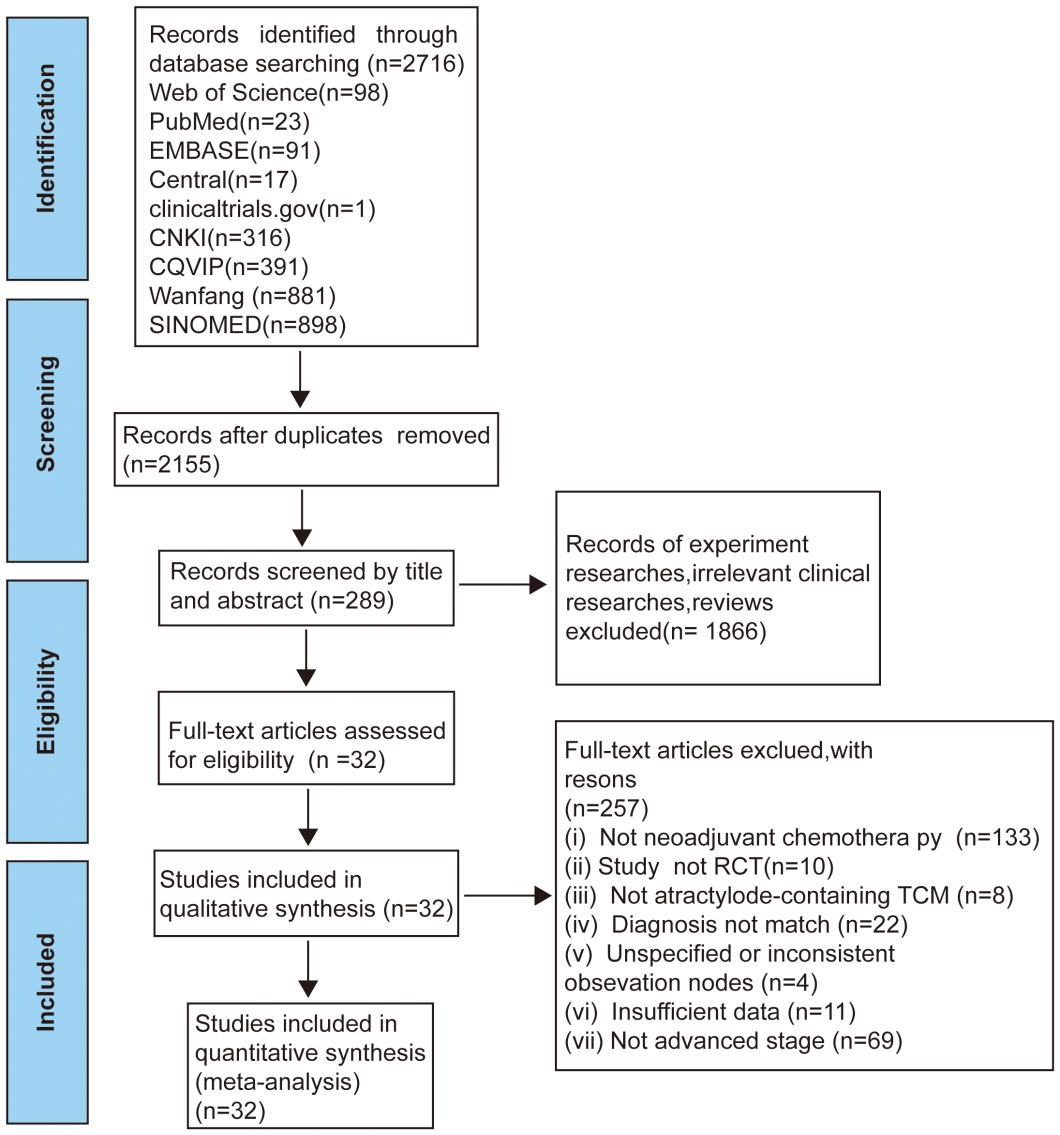
Flow diagram of selection process (17).

In total 2716 records identified via comprehensive database searches. Total records underwent screening comments from two reviewers through a process consisting of three steps. Firstly, We reviewed titles and eliminated duplicate records 2155 records. Secondly, we included 289 full-text articles by reading the titles or abstracts. Thirdly, we evaluated the full text was assessed based on the incorporation of and exclusion criteria and excluded 257 trials. Finally, 32 eligible studies involving 2157 patients with stage II-IV AGC were incorporated into our study (**Figure 1**).

### 3.2 Characteristics of the included studies

Details regarding the features included in the study are presented in **Table 1**. All 32 studies were conducted in China. A total of 1383 male patients and 774 female patients, with sample size ranging from 30 to 135. All clinical trials were founded on the main drug of Atractylodes macrocephala-containing the traditional Chinese medicine in combination with neoadjuvant chemotherapy.

**Table 1:** Characteristics of the included studies.

Study	Advanced stage gastric cancer				Interventions			Follow up	Criteria	Outcome
	TP	E/C	M/F	TNM stage	Atractylodes-containing TCM	Drug delivery	Neoadjuvant chemotherapy regimen			
Ma.2020 (18)	Unclear	15/15	17/13	IV	Atractylodis macrocephala, Pinellia ternate 、Radix pseudostellariae 、Curcuma zedoary 、Coix seed 、Citrus reticulata 、Astragalus 、Hericium erinaceus 、Polygonatum odoratum、Silkworm pupa,400ml/d,8w	Orally	S-1:100mg,d1-14;OXA:100 mg/m^2^,d1;3W/C, 2cycles	8w	RECIST	O1,2
Sun et al.2013 (19)	Unclear	20/20	25/15	III,IV	Atractylodis macrocephala, Astragalus, Codonopsis, Poria cocos, Chimonanthus praecox, Perilla Stem, Dioscoreae opposita, Coix seed, Angelica sinensis, Paeonia lactiflora, Polygonatum odoratum, 400ml/d,8w	Orally	S-1:80mg/m^2^, d1-28,4W/C,2cycles	8w	RECIST,WHO	O1,2,3,4,5
Wang.2018 (20)	TP	21/21	26/16	IV	Atractylodis macrocephala、Astragalus、Hedyotis diffusa、Radix pseudostellariae 、Codonopsis、Hericium erinaceus、Dandelion、Radix salviae miltiorrhizae 、Pinellia ternate、Chimonanthus praecox、Trichosanthes kirilowii、Curcuma zedoary、Citrus reticulata,400ml/d,16W-24W	Orally	S-1:80mg,d1-14;OXA:130mg/m^2^,d1;3W/C, 4-6cycles	16W-24W	/	O1,2,4
Li.2021 (21)	Unclear	21/21	21/21	I,II,III,IV	Atractylodis macrocephala、Astragalus、Saxifraga stolonifera、Curcuma zedoary、Curcuma zedoary、Lycium barbarum、Epimedium、Codonopsis、Poria cocos、Morinda officinalis、Cistanche deserticola、Beeswax, 300ml/d,24w	Orally	OXA:130 mg/m^2^,d1;CAP:850-1250 mg/m^2^,14 d;3W/C, 6cycles	24W	CSCO	01,2,4,5
Xu,W.2020 (22)	Unclear	26/26	29/23	II,III,IV	Atractylodis macrocephala、Citrus reticulata、Radix pseudostellariae 、Polygonatum odoratum、Astragalus、Hericium erinaceus、Pinellia ternate、Silkworm pupa、Curcuma zedoary、Coix Seed, 400ml/d,6W	Orally	S-1:100mg,d1-14;OXA:100 mg/m^2^,d1;3W/C, 2cycles	6W	/	O1,2,3
Yan et al.2019 (23)	TP	28/28	31/25	IV	Atractylodis macrocephala、Codonopsis、Glycyrrhiza、Rehmannia glutinosa、Paeonia lactiflora、Poria cocos、Ligusticum chuanxiong、Angelica sinensis, 400ml/d,6W	Orally	DOC:75 mg/m^2^, d1; DDP: 20 mg/ m^2^,d1–5; 5-Fu:1000 mg/m^2^, d1–5;28d/C, 2 cycles	6-8W	RECIST,NCI	O1,2,3,4
Ye et al.2017 (24)	Unclear	30/30	36/24	II:23, III:20, IV:17	Atractylodis macrocephala、Codonopsis、Astragalus、Cimicifuga foetida、BupLVrum、Citrus reticulata、Angelica sinensis 、Curcuma zedoary、Ophiopogon japonicus、Rhizoma Imperatae、Hedyotis diffusa、Acalypha australis、Rhizoma Anemarrhenae、Ligustrum lucidum ait、Malt 、Ziziphus jujuba、Glycyrrhiza,400ml/d,8W	Orally	OXA:130mg/m^2^,d1;XELO-DA:100 mg/m^2^,d1-4,3W/C, 2cycles	6-8W	RECIST	O1,2,3
Zhu.2019 (25)	Unclear	30/30	35/25	III,IV	Atractylodis macrocephala、Astragalus、Codonopsis、Angelica sinensis 、Glycyrrhiza、Citrus reticulata、Cimicifuga foetida、BupLVrum、Curcuma zedoary、Ophiopogon japonicus、Rehmannia glutinosa、Polygonatum odoratum、Zingiber officinale、Ziziphus jujuba,400ml/d,	Orally	OXA:130 mg/m²,d1;S-1:40 mg/m^2^, d1-14, 3W/C,2cycles	8w	RECIST	O1,2,3
Wu et al.2020 (26)	Unclear	30/30	38/22	III	Atractylodis macrocephala、Angelica sinensis 、Ligusticum chuanxiong、Paeonia lactiflora、Rehmannia glutinosa、Codonopsis、Poria cocos、Glycyrrhiza、Pinellia ternate、Poria cocos、Coix seed、Astragalus、Amomum villosum,400ml/d,9W	Orally	OXA:100 mg/m^2,^ ,d1;S-1:BS < 1.25 m^2^,80 mg/d,1.25-1.5 m^2^,100 mg/d, > 1.5m^2^,60mg/d,14d,3W/C,3cycles	9w	RECIST,WHO	01,2,3,5
Zhang et al.2005 (27)	Unclear	30/30	38/22	III,IV	Atractylodis macrocephala、Astragalus、Paeonia lactiflora、Cynanchum stauntonii、Black Cardamom、Sepiae Os、Poria cocos、Glycyrrhiza、Ostrea gigas Thunberg、Lapis Chloriti、Commiphora myrrha、Angelica sinensis 、Chimonanthus praecox、Notoginseng radix、Panax quinquefolius,400ml/d,12W	Orally	CF:200mg/d,d1-5;5-Fu:500mg/d,d1-5,3W/C,3cycles	12W	RECIST,WHO	O1,2,3
Hu.2009 (28)	Unclear	30/30	47/13	IV	Atractylodis macrocephala、Codonopsis、Poria cocos、Coix seed、Poria cocos、Poria with Hostwood、Dioscoreae opposita、Folium Eriobotryae、Germinatus Hordei Vulgaris、Malt、Citrus reticulata、Pinellia ternate, 90ml/d,16-24W	Orally	OXA:130 mg/m^2^,d1-14;5-Fu:400mg/m^2^,d1-14;LV:200mg/m^2^,d1-14,2W/C, 6cycles	16-24W	WHO	O1,2,3
Huang,C.2014 (29)	Unclear	30/30	33/27	IV	Atractylodis macrocephala、Codonopsis、Radix pseudostellariae 、Dandelion、Perilla Stem、Piper longum、Trichosanthes kirilowii Maxim、Pinellia ternate、Gekko gecko、Curcuma zedoary、Crataegus pinnatifida、Rheum palmatum、Magnolia bark、Chimonanthus praecox, 400ml/d,8W	Orally	OXA:85 mg/m^2^,d1;5-Fu:1000mg/m^2^,d1-2;LV:200mg/m^2^,d1-2,2W/C, 2cycles	8w	WHO	O1,2,3,4
Wang,L.2015 (30)	TP	30/30	44/16	III,IV	Atractylodis macrocephala、Codonopsis、Astragalus、Poria cocos 、Dioscoreae opposita 、Lycium barbarum 、Paeonia lactiflora、Cornus officinalis、Angelica sinensis 、Ligustrum lucidum ait、Achyranthes bidentata、Glycyrrhiza, 300ml/d,6W	Orally	EPI:50 mg /m^2^ d1;DDP:60 mg /m^2^,d1;CAP:1000 mg/m^2^,d1-14,3 W/C,2cycles	6W	RECIST,WHO	01,2,3,4,5
Guo.2016 (31)	Unclear	34/34	40/28	III,IV	Atractylodis macrocephala、Poria cocos、Astragalus、Coix seed、Panax ginseng、Semen Lablab Album、Dioscoreae opposita、Lotus Plumule, Amomum villosum、Platycodon grandiflorus、Glycyrrhiza, 400ml/d,12W	Orally	OXA:130mg/m^2^,d1;5-Fu:750mg/m^2^,d1-5;LV:200mg/m^2^,d1-5,3W/C, 4cycles	12W	WHO	O1,2,4
Zhao.2018 (32)	Unclear	38/38	43/33	III,IV	Atractylodis macrocephala、Astragalus、Coix seed、Semen Lablab Album、Dioscoreae opposita、Poria cocos、Lotus Plumule、Codonopsis、BupLVrum、Cimicifuga foetida、Angelica sinensis 、Citrus reticulata, 250ml/d,12W	Orally	PTX: 175 mg/m^2^, d1; DDP: 25 mg/m^2^, d 1–5; 5-Fu: 600 mg/m^2^, d 5–9; 28d/C, 2cycles	8w	/	O1,2
Zhang.2017 (33)	Unclear	40/40	53/28	IV	Atractylodis macrocephala、Poria cocos、Codonopsis、Dioscoreae opposita、Atractylodis macrocephala、Citrus reticulata、Coix seed、Paeonia lactiflora、Lotus Plumule、Pinellia ternate、Semen Lablab Album、Amomum villosum、Angelica sinensis 、Platycodon grandiflorus、Costusroot、Saxifraga stolonifera、Hedyotis diffusa、Curcuma zedoary、Trichosanthes kirilowii 、Glycyrrhiza, 400ml/d,8W	Orally	PTX:75mg/m^2^,d1-8;S-1:80mg/m^2^,d1-14,2W/C,4cycles	8w	RECIST,NCI	O1,2,3,4
Li et al.2019 (34)	TP	40/40	42/38	I,II,III,IV	Atractylodis macrocephala、Paeonia lactiflora、Codonopsis、Coix seed、Curcuma zedoary、Cinnamomum twig、Saxifraga stolonifera、Forsythia suspensa、Herba Lysimachiae、Hedyotis diffusa、scorpion、Glycyrrhiza, 400ml/d, 6W	Orally	LV:200 mg /m^2^, d1-5;5-Fu:600 mg /m^2^, d1-5;EPI:50mg /m^2^,d1;DDP:20 mg /m^2^, d1-5,3W/C,3cycles	6W	RECIST,WHO	01,2,3,4,5
Wu,C.2017 (35)	Unclear	40/40	48/32	IIIA,IIIB,IV	Atractylodis macrocephala、Curcuma zedoary、Poria cocos、Amaranthus mangostanus、Coix Seed、Hedyotis diffusa、Semen Lablab Album、Citrus reticulata、Pinellia ternate、Trichosanthes kirilowii、Citrus medica var. sarcodactylis、Glycyrrhiza、Ophiopogon japonicus、Malt、Germinatus Hordei Vulgaris, 300ml/d,18W	Orally	OXA:130 mg/m²,d1;S-1:80 mg/m^2^, d1-14, 3W/C,6cycles	18W	WHO	O1,2
Wei et al.2018 (36)	Unclear	42/42	66/18	IV	Atractylodis macrocephala、Panax ginseng、Poria cocos、Glycyrrhiza、Angelica sinensis 、Rehmannia glutinosa、Ligusticum chuanxiong、Paeonia lactiflora,400ml/W,6w	Orally	OXA:150mg/m^2^,d1;5-Fu:1500mg/m^2^,d1;LV:300mg/m^2^,d1-2,2W/C, 3cycles	6W	WHO,NCI	O1,2
Wen et al.2021 (37)	TP	45/45	56/34	IIB,IIIA,IIIB	Atractylodis macrocephala、Astragalus、Radix pseudostellariae 、Dioscoreae opposita、Poria cocos、Coix seed、Hedyotis diffusa、Ganoderma lucidum、Radix salviae miltiorrhizae 、Ligusticum chuanxiong、Curcuma zedoary、Herba Lysimachiae、Aconitum carmichaelii Debeaux、Ophiopogon japonicus、Dictamnus dasycarpus、Glycyrrhiza, 300ml/d,6W	Orally	LV:400mg/m^2^,d1;OXA:85mg/m^2^,d2;5-Fu:2400mg/m^2^,d1-2,2W/C, 3cycles	6W	RECIST,NCI	O1,2,4,5
Zhang,L.2021 (38)	Unclear	45/45	61/29	IIIA,IIIB,IV	Atractylodis macrocephala、Astragalus、Codonopsis、Rehmannia glutinosa、Curcuma zedoary、Ophiopogon japonicus、Citrus reticulata、BupLVrum、Polygonatum odoratum、Angelica sinensis 、Curcuma zedoary、Hedyotis diffusa、Glycyrrhiza, 400ml/d,8W	Orally	DOC:40mg/m^2^,1W/^-1^,6W;OXA:130mg/m^2^,3W/^1^;CAP:2500mg/d,d1-14,8W/C.1cycles	8w	RECIST	O1,2
Li et al.2016 (39)	TP	50/50	55/45	III,IV	Atractylodis macrocephala、Codonopsis 、Poria cocos、Dioscoreae opposita、Coix seed、Citrus reticulata、Lotus Plumule 、Pinellia ternate、Paeonia lactiflora、Semen Lablab Album、Angelica sinensis 、Amomum villosum、Costusroot、Platycodon grandiflorus、Hedyotis diffusa、Saxifraga stolonifera、Trichosanthes kirilowii、Curcuma zedoary、Glycyrrhiza, 400ml/d,8W	Orally	PTX: 75 mg/m^2^, d1,8; S-1:160mg/ m^2^, d1-14 4W/C, 2cycles	8w	RECIST,NCI	O1,2,3,4
Zhu et al.2019 (40)	Unclear	61/61	62/60	IIIB,IV	Atractylodis macrocephala、Radix pseudostellariae 、Astragalus、Poria cocos、Rehmannia glutinosa、Angelica sinensis 、Semen Cuscutae、Lycium barbarum、Centella asiatica、Atractylodes macrocephala、Citrus medica var. sarcodactylis、Polygonatum odoratum、Hedyotis diffusa、Glycyrrhiza, 400ml/d,8W	Orally	OXA:85mg/m^2^,d1;CF:200mg/m^2^,d1-2;5F-u:1000mg/m^2^,d1-2,2W/C,4cycles	8W	/	O1,2,5
Xie et al.2021 (41)	Unclear	29/27	45/11	I,II,IIIA,IIIB,IIIC	Atractylodis macrocephala、Astragalus、Codonopsis、Angelica sinensis 、Paeonia lactiflora、Citrus reticulata、Pinellia ternate 、Trichosanthes kirilowii、Curcuma zedoary、Saxifraga stolonifera、Hedyotis diffusa、Glycyrrhiza, 400ml/d,6W	Orally	OXA:130mg/m^2^,d1;S-1:80mg/m^2^,d1-14;3W/C, 2cycles	6W	/	O1,2
Xie et al.2022 (42)	TP	32/28	50/10	II,III	Atractylodis macrocephala、Astragalus、Codonopsis、Poria cocos、Glycyrrhiza, 400ml/d,4W	Orally	PTX:50mg/m^2^,d1;OXA:85mg/m^2^,d1;CF:400mg/m^2^,d1;5-Fu:1200mg/m^2^,d1,2W,2cycles	4W	/	O4
Li.2011 (43)	Unclear	27/29	33/23	III,IV	Atractylodis macrocephala、Astragalus、Codonopsis、Poria cocos、Lotus Plumule、Dioscoreae opposita、Semen Lablab Album、Angelica sinensis 、Cimicifuga foetida、BupLVrum、Coix seed、Citrus reticulata, 250ml/d,12W	Orally	DDP:20 mg/m^2^, d1-5; 5-Fu: 750 mg/m^2^, d1-5,4W/C,3cycles	12W	/	O1,2
Zheng et al.2012 (44)	Unclear	35/30	56/9	IIIB,IV	Atractylodis macrocephala、Radix pseudostellariae 、Astragalus、Poria cocos、Pinellia ternate、Radix scutellariae、Rhizoma Coptidis、Cinnamomi Ramulus、Radix Codonopsis、Curcuma zedoary、Coix seed、Citrus reticulata、Fritillariae Bulbus、Aristolochia contorta Bge.、Lablab purpureus、Glycyrrhiza, 300ml/d,8W	Orally	CF:100mg/m^2^,d1-5;5-Fu:500mg/d,d1-5;OXA:130mg/m^2^,d1,3W/C,2cycles	8W	WHO	O1,2,3
Hu et al.2010 (45) Zhu,Q.2013 (46) Lai et al.2010 (47)	Unclear Unclear TP	28/32 24/22 25/30	36/24 29/17 44/11	IV III,IV IV	Atractylodis macrocephala、Codonopsis、Curcuma zedoary、Saxifraga stolonifera、Astragalus、Dioscoreae opposita、Poria cocos、Radix Rehmanniae、Ophiopogon japonicus、Corydalis yanhusuo、Pinellia ternate、Citrus reticulata、Glycyrrhiza, 400ml/d,8W Atractylodis macrocephala、Codonopsis、Poria cocos、Angelica sinensis 、Cimicifuga foetida、Dioscoreae opposita、Lotus Plumule、BupLVrum、Semen Lablab Album、Astragalus、Amomum villosum、Coix seed、Citrus reticulata、Platycodon grandiflorus、Glycyrrhiza Atractylodis macrocephala、Astragalus、Codonopsis、Poria cocos、Dioscoreae opposita、Lycium barbarum、Cornus officinalis、Achyranthes bidentata、Paeonia lactiflora、Angelica sinensis 、Ligustrum lucidum ait、Glycyrrhiza, 300ml/d,8W	Orally Orally Orally	OXA:130mg/m^2^,d1;LV:100mg/m^2^,d1-5;5-Fu:300mg/m^2^,4W/C,2cycles DOC:75mg/m^2^, d1,DDP:75mg/m^2^, d1-2, 5-Fu:500mg/m^2,^d1-5, 3W/C,2cycles PTX:100 mg/m^2^,d1;5-FU 2.0g/m^2^,d1;CF:0.3g/m^2^, d1-2,2W/C,4cycles	8w 6W 8w	RECIST / WHO,WHO	O1,2 O1,2,4 O1,2
Li,S.2014 (48)	Unclear	40/32	38/34	IIIB,IIIC,IV	Atractylodis macrocephala、Codonopsis、Astragalus、Poria cocos、Angelica sinensis 、Rehmannia glutinosa、Paeonia lactiflora、Acalypha australis、Semen Cuscutae、Herba Spiraeae Thunbergii、Ligustrum lucidum ait、Colla Corii Asini、Coix seed、Germinatus Hordei Vulgaris、Malt、Ziziphus jujuba、Zingiber officinale、Glycyrrhiza, 400ml/d,8W	Orally	OXA:130mg /m^2^, d1;XELO-DA:200mg /m^2^,d1-14,3W/C,2cycles	8w	RECIST	O1,2,3
Chen et al.2013 (49)	Unclear	84/51	96/39	IIIA,IIIB,IIIC,IV	Atractylodis macrocephala、Codonopsis、Poria cocos、Dioscoreae opposita、Coix seed、Citrus reticulata、Costusroot、Angelica sinensis 、Paeonia lactiflora、Caulis Spatholobi、Saxifraga stolonifera、Glycyrrhiza, 450ml/d,12W	Orally	DOC:75mg/m^2^, d1,DDP:75mg/m^2^, d1-2, 5-Fu:500mg/m^2^,d1-5, 3W/C,4cycles	12W	/	O3

E/C, experimental group (white herbal medicine containing NAC)/control group (NAC alone); NAC, neoadjuvant chemotherapy; M / F, male/female; for investigator, 5-Fu, 5-fluorouracil; OXA, oxaliplatin; PTX, paclitaxel; DDP, cisplatin; CF, Calcium folinate; DOC, docetaxel; CAP, capecitabine; WHO, World Health Organization guidelines for solid tumor responses; RECIST, Response Evaluation Criteria in Solid Tumors; \, unclear; O, outcome; O1, objective response rate (ORR); O2, disease control rate (DCR); O3, quality of life (QOL); O4, adverse drug reactions (ADRs); O5,peripheral blood lymphocyte levels.

Conclusions: The specific information of 32 gastric cancer patients in literature was included, including age, sex, number of patients, tumor stage, Atractylodes macrocephala-containing traditional Chinese medicine combined ingredient, follow-up time, chemotherapy regimen, tumor evaluation criteria, etc.

Follow-up ranged from 4 W to 24 W, tumor responses were reported in 7 trials according to the WHO guidelines and in 15 trials according to the RECIST guidelines. Adverse effects were reported in 6 trials using the WHO.NCI CTCAE criteria were used in 5 cases, and unknown criteria were used in 9 cases.

### 3.3 Risk of bias

**Figure 2:**
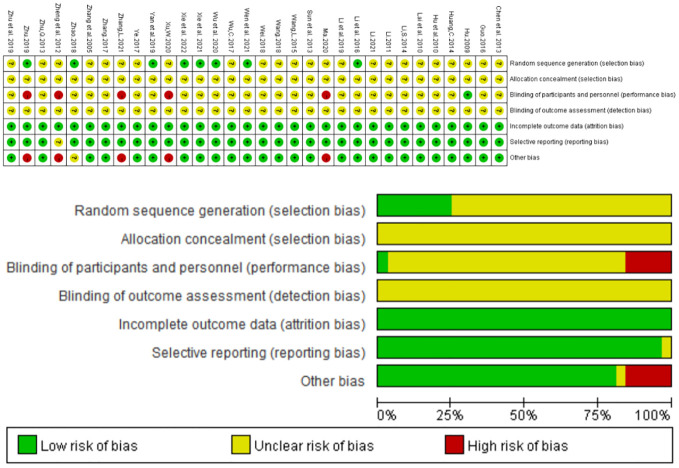
Assessment of methodological bias risk.

32 studies involved randomization; Of these, eight studies (24, 26, 32, 34, 37, 40–42) used random number tables to generate random sequences, three studies used admission order (18, 25, 44), and two studies (22, 38) used treatment modalities, none of which adequately described masking for investigators, patients, and outcome evaluators. The exposure bias for each individual trial is summarized in **Figure 2** below.

### 3.4 Tumor response

**Figure 3:**
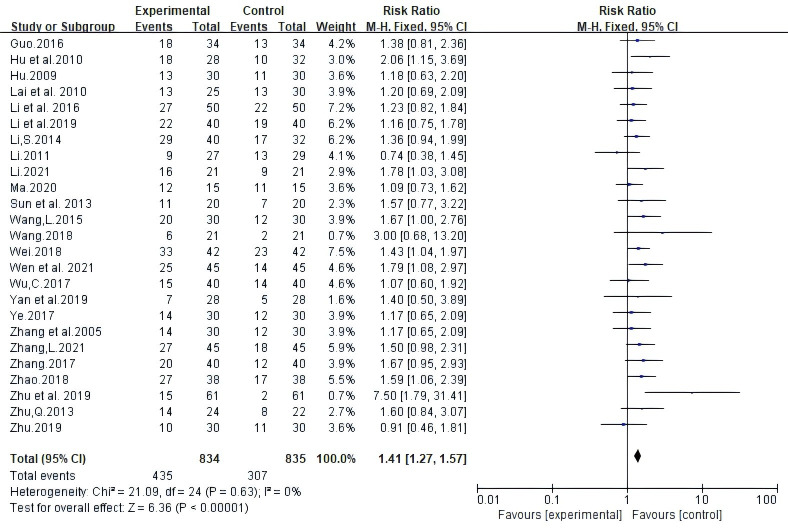
Meta-analysis results of objective response rate (ORR) between the two groups.

Twenty-five trials involving 1669 patients reported ORR according to Guidelines from the World Health Organization (WHO) or Response Evaluation Criteria In Solid Tumors (RECIST). Random-effects meta-analysis demonstrated that White Atractylodes Chinese herbal medicine combined with NAC improved ORR; the difference was statistically significant (RR: 1.41, 95% CI: 1.27 ~ 1.57, P < 0.00001, I^2^ = 0%, **Figure 3**).

**Figure 4:**
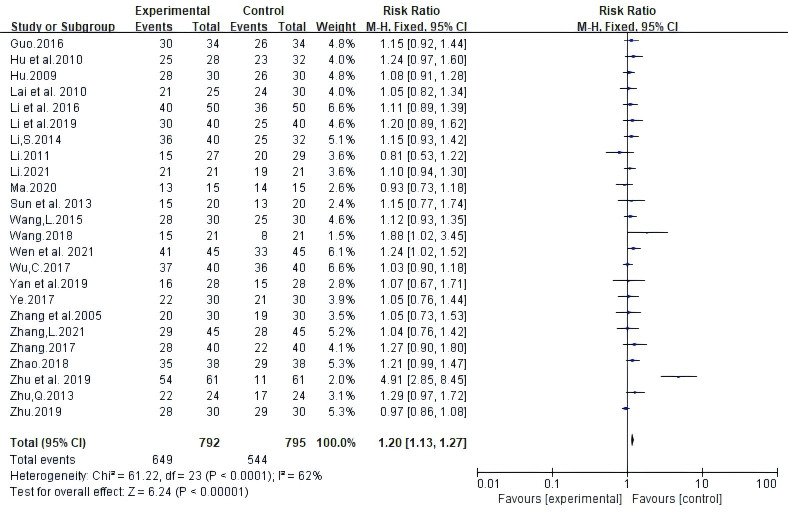
Meta-analysis results of disease control rate (DCR) between the two groups.

Twenty-four test reports involved 1,587 DCR. Meta-analysis using random-effects models demonstrated that the combination of Atractylodes macrocephala and NAC enhanced Disease Control Rate DCR; statistically significant differences (RR: 1.20, 95% CI: 1.13 ~ 1.27, P < 0.00001, I^2^ = 62%, **Figure 4**).

### 3.5 Quality of life

**Figure 5:**
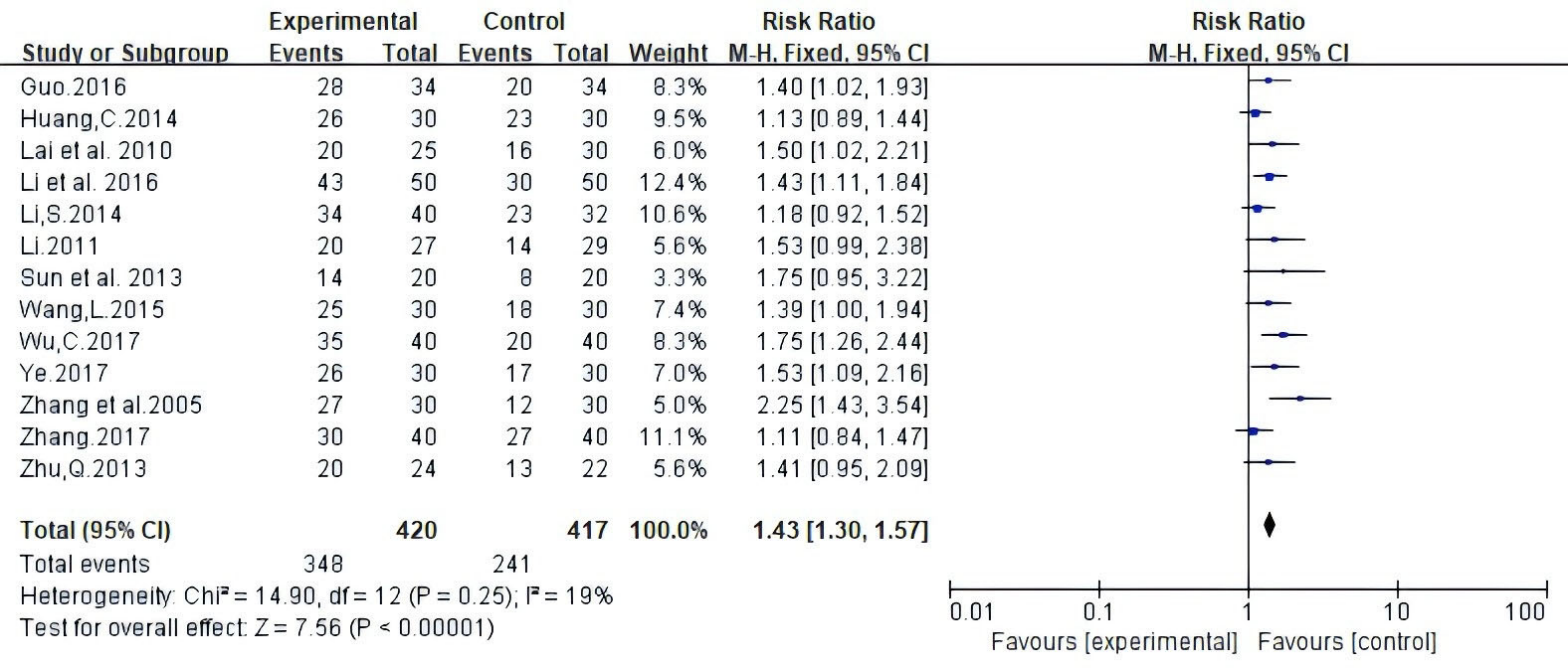
Meta-analysis results of quality of life (QOL) according to the number of KPS improved patients.

**Figure 6:**
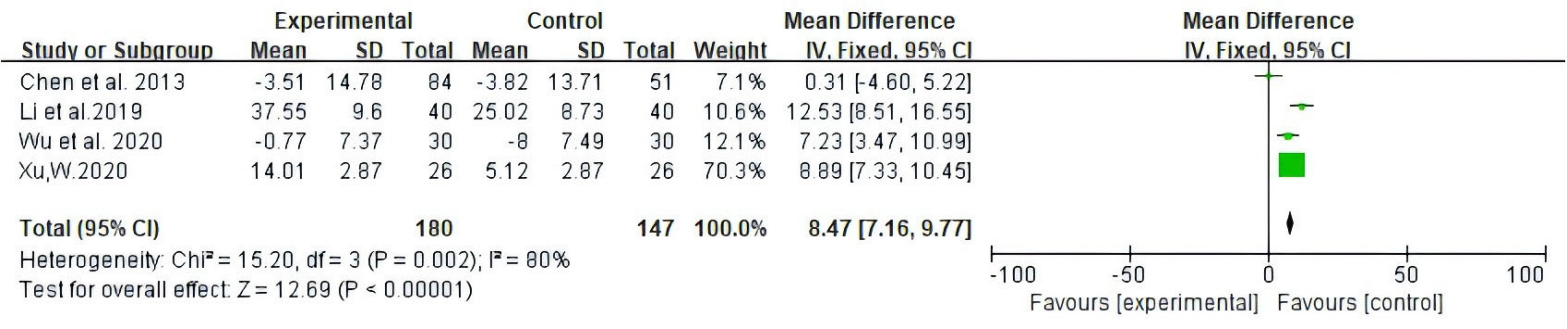
Meta-analysis results of QOL according to mean ± SD.

Among the studies covered, two forms of data were used to document improvements in quality of life based on the assessment of KPS scores. The first is number of patients indicating improvement in quality of life. Secondly, the mean ± standard deviation (SD) of KPS scores pre- and post-treatment were reported. As illustrated in **Figure 5**, 13 studies reported patient count with improved quality of life after the KPS scores (RR: 1.43, 95% CI: 1.30-1.57, P = 0.25, I^2^ = 19%); the other four studies all reported the mean ± SD of KPS scores (MD: 8.47, 95% CI: 7.16 - 9.77, P = 0.002, I^2^ = 80%) (**Figure 6**). In conclusion, compared with NAC, Atractylodes macrocephala-containing with NAC substantially enhanced the patients’ quality of life.

### 3.6 Adverse drug reactions

**Figure 7:**
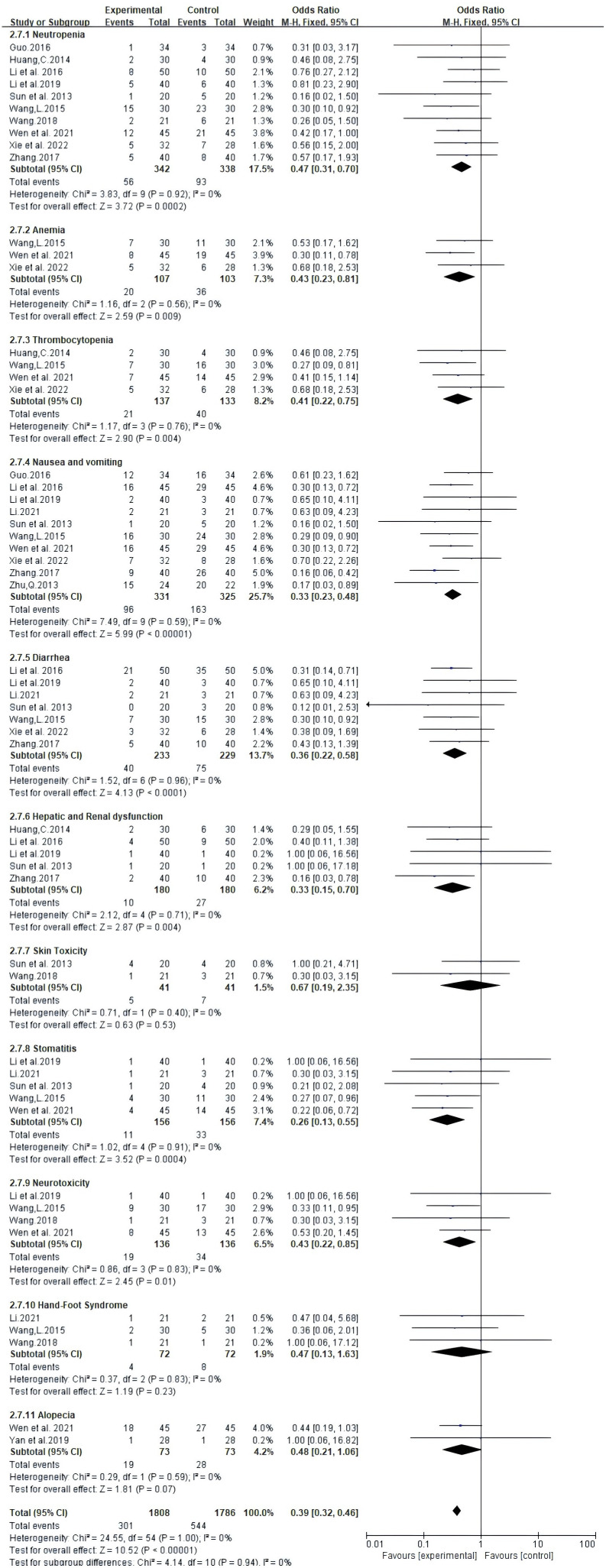
Meta-analysis results of adverse drug reactions (ADRs) between the two groups.

Adverse effects reported in 13 trials that included 914 patients mainly met the WHO criteria (**Table 2**, **Figure 7**). The findings from Meta-analysis with random effects models indicated that when compared to NAC used independently, the combination of Atractylodes macrocephala-containing herbs with NAC significantly reduced leukocytosis (RR: 0.60, 95% CI: 0.45-0.79, P = 0.0002), anemia (RR: -0.2, 95% CI: -0.34–0.06, P = 0.006), thrombocytopenia (RR. 0.51, 95% CI: 0.32-0.81, P = 0.005), nausea and vomiting (RR: 0.33, 95% CI: 0.23-0.48, P < 0.00001), diarrhea (RR: 0.53, 95% CI: 0.39 - 0.72, P < 0.0001), and liver and renal dysfunction (RR: 0.37, 95% CI. 0.19 - 0.74,P=0.005), danger of neurotoxicity (RR: 0.43, 95% CI: 0.22 ~ 0.85, P = 0.01), stomatitis (RR: 0.26, 95% CI: 0.13 ~ 0.55, P = 0.0004), but incidence of alopecia (RR: 0.68, 95% CI: 0.44 ~ 1.04, P = 0.08), hand-foot syndrome (RR: 0.68, 95% CI: 0.13 ~ 1.63, P = 0.23) were not associated.

**Table 2:** Meta-analysis results of adverse drug reactions (ADRs).

Outcomes	Trials	Experimental group (Events/Total)	Control group (Events/Total)	SM	RR, 95%CI	I^2^	P
Neutropenia	10	342	338	REM	0.47 [0.31, 0.70]	0%	0.0002
Anemia	3	107	103	REM	0.43 [0.23, 0.81]	0%	0.009
Thrombocytopenia	4	137	133	REM	0.41 [0.22, 0.75]	0%	0.004
Nausea and vomiting	10	331	325	REM	0.33 [0.23, 0.48]	0%	<0.00001
Diarrhea	7	233	229	REM	0.36 [0.22, 0.58]	0%	<0.0001
Hepatic and Renal dysfunction	5	180	180	REM	0.33 [0.15, 0.70]	0%	0.004
Skin Toxicity	2	41	41	REM	0.67 [0.19, 2.35]	0%	0.53
Stomatitis	5	156	156	REM	0.26 [0.13, 0.55]	0%	0.0004
Neurotoxicity	4	136	136	REM	0.43 [0.22, 0.85]	0%	0.01
Hand-Foot Syndrome	3	72	72	REM	0.47 [0.13, 1.63]	0%	0.23
Alopecia	2	73	73	REM	0.48 [0.21, 1.06]	0%	0.07

RR, risk ratio; CI, confidence interval; SM, statistical method; REM, random-effect model.

Conclusions : The side effects of Atractylodes macrocephala-containing traditional Chinese medicine combined with neoadjuvant chemotherapy were less than that of chemotherapy alone.

### 3.7 Peripheral blood lymphocyte levels

**Figure 8:**
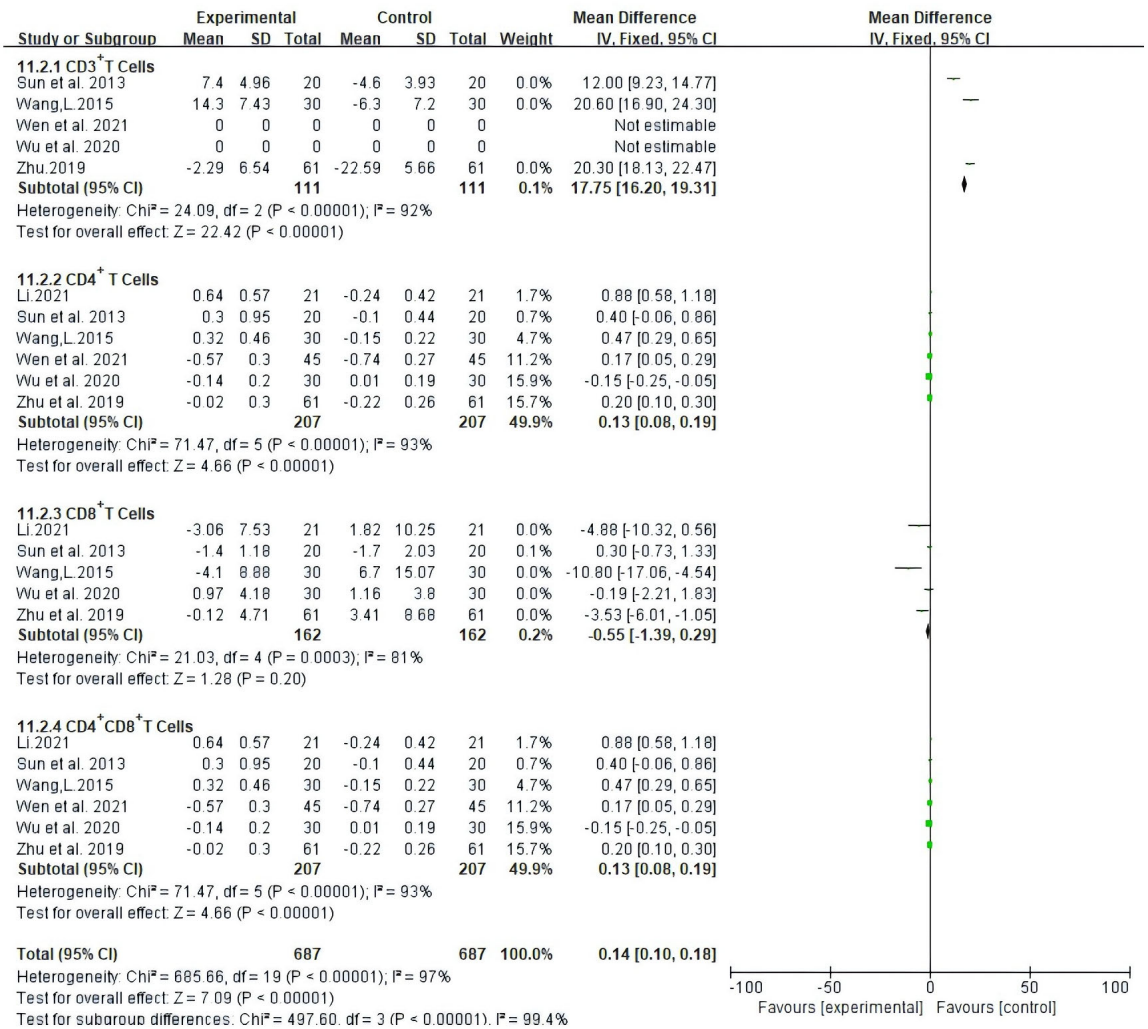
Meta-analysis results of Meta-analysis results of CD3+ T cells,CD4+ T cells, CD4+ CD8+ Tcells between the two groups.

Seven trials, involving 494 patients, documented the levels of peripheral blood lymphocytes. Our results showed that Atractylodes macrocephala-containing herbs plus NAC raise significantly CD3^+^ T cells (MD: 17.75, 95% CI: 16.2-19.31, P < 0.00001), CD4^+^ T cells (MD: 0.13, 95% CI: 0.08-0.19, P < 0.00001), CD8^+^ T cells (MD:-0.55, 95% CI: -1.39-0.29, P < 0.00001) and the proportion of CD4^+^CD8^+^ T cells (MD: 0.13, 95% CI: 0.08-0.19, P < 0.00001) (**Figure 8**).

### 3.8 Subgroup analysis

Our subgroup analyses were performed according to the treatment procedures, the NAC regimen, treatment duration, and inclusion of Atractylodes macrocephala-containing herbs were examined to assess their impact on the objective response rate ORR and disease control rate DCR. Individuals were categorized into primary treatment (PT) and unspecified treatment according to treatment procedures. Subgroup analyses showed that atractylodes-containing herbs improved ORR and DCR in both primary and indeterminate treatment (**Table 3**).The NAC regimen was divided into two subgroups, fluorouracil-based and platinum-based. The fluorouracil subgroup was further divided into three chemotherapy regimens based on 5-Fu, S-1,CAP. The platinum-based subgroup was further divided into two chemotherapy regimens based on DDP and OXA.The subgroup findings suggested that regardless of the chemotherapy regimen with 5-Fu, S-1,CAP, OXA, and DDP, the Atractylodes macrocephala-containing herbs improved ORR and DCR, except for the use of cisplatin, which was not associated with DCR. two independent duration of treatment were established: <8 weeks and ≥8 weeks. According to the subgroup analysis, regardless of duration of treatment of <8 weeks or ≥8 weeks, leucovorin-containing herbal medicines improved ORR and DCR.

**Table 3:** Subgroup analysis of ORR and DCR.

Subgroups	Number of studies	RR (95% CI)	Z	P	Heterogeneity	
					I^2^	P_h_
1.Objective response rate (ORR)						
Therapy procedure						
therapy procedure (PT)	7	1.95[1.34, 2.82]	3.52	P=0.0004	0%	0.80
Unclear	18	1.98[.55, 2.52]		P<0.00001	12%	0.31
use of fluoropyrimidine						
S-1-based chemotherapy regimen	7	1.56[1.05, 2.33]	2.21	P=0.03	0%	0.70
5 Fu-based chemotherapy regimen	12	1.87 [1.40, 2.49]	4.26	P<0.0001	2%	0.47
CAP-based chemotherapy regimen	3	2.80 [1.56, 5.04]	3.43	P=0.0006	0%	0.72
use of platinum						
DDP-based chemotherapy regimen	6	1.75 [1.15, 2.66]	2.62	P=0.009	24%	0.26
OXA-based chemotherapy regimen	14	2.17 [1.65, 2.86]	5.49	P<0.00001	8%	0.36
Treatment duration						
<8 weeks	7	2.04 [1.40, 2.96]	3.72	P=0.0002	0%	0.80
≥8 weeks	18	2.05 [1.61, 2.62]	5.74	P<0.00001	11%	0.32
2.Disease control rate (DCR)						
Therapy procedure						
therapy procedure (PT)	7	1.95 [1.32, 2.87]	3.37	P=0.0008	0%	0.74
Unclear	17	1.92 [1.49, 2.47]	5.10	P<0.00001	13%	0.3
use of fluoropyrimidine						
S-1-based chemotherapy regimen	7	1.56 [1.05, 2.33]	2.21	P=0.03	0%	0.70
5 Fu-based chemotherapy regimen	11	0.14 [0.07, 0.21]	3.84	P=0.0001	5%	0.40
CAP-based chemotherapy regimen	3	2.80 [1.56, 5.04]	3.43	P=0.0006	0%	0.72
use of platinum						
DDP-based chemotherapy regimen	6	1.37 [0.86, 2.17]	1.32	P=0.19	11%	0.34
OXA-based chemotherapy regimen	13	2.97 [2.11, 4.18]	6.22	P<0.00001	67%	0.0003
Treatment duration						
<8 weeks	6	1.89 [1.26, 2.84]	3.06	P=0.002	0%	0.81
≥8 weeks	18	2.05 [1.61, 2.62]	5.74	P<0.00001	11%	0.32

RR, risk ratio; CI, confidence interval; DDP, cisplatin; OXA, oxaliplatin;S-1,Tegafur,Gimeracil and Oteracil Potassium Capsules;5-Fu,5-fluorouracil;CAP,capecitabine.

Conclusion: The ORR and DCR with Atractylodes macrocephala-containing traditional Chinese medicine with neoadjuvant chemotherapy are more effective than those with chemotherapy alone.

As previously mentioned (50, 51), we performed a subgroup analysis of specific ingredients containing Atractylodes macrocephala preparations in each literature to determine the contribution of the combination of Atractylodes macrocephala and other herbs to advanced gastric cancer. **Tables 4** and **5** include only combinations of Chinese medicines with low heterogeneity (I^2^ < 30%). In 32 literatures, a total of 90 TCM were used, with an average of 13 TCM. Among the most commonly used traditional Chinese medicines in combination with Baizhou in the treatment of advanced gastric cancer, the following are Codonopsis(n=24),Astragalus(n=23),Poria cocos(n=23),Glycyrrhiza(n=23) and Angelica sinensis(n=19),Curcuma zedoary(n=18),Coix seed(n=17). When these plants were present in combinations of pairs, triples, and higher levels in the TM intervention, their effects on ORR and DCR were evaluated, and when TMs combinations showed higher RRs than TMs alone, these were considered possible examples of synergies. **Table 4** shows that at Level 1, when Baichu is combined with other single Chinese medicine, the most commonly used combination is Atractylodis macrocephala+Poria cocos (n=19; RR=1.98 [1.57, 2.49],I^2^ = 0), Atractylodis macrocephala+Malt (n=4; RR=1.45 [0.89, 2.36], I^2^ = 0) was the lowest, and RR of the 10 combinations were significantly higher than that of the total pool (1.41). Level 2, combinations of 3 TMs, the most common combinations are Atractylodis macrocephala+Poria cocos+Glycyrrhiza (n=14; RR=2.18 [1.68, 2.84], I^2^ = 0), the lowest RR of Atractylodis macrocephala+Panax ginseng+Astragalus(n=2) was 1.56 [0.77, 3.14]. RR of 19 TCM combinations was significantly higher than that of total pool (1.41). Level 3, combinations of 4 TMs, the most common combinations are Atractylodis macrocephala+Codonopsis+Astragalus+Angelica sinensis (n=10; RR=1.77 [1.29, 2.45], I^2^ = 2),Atractylodis macrocephala+Poria cocos+Glycyrrhiza+Angelica sinensis (n=10; RR=2.22 [1.61, 3.06], I^2^ = 0), while Atractylodis macrocephala+Codonopsis+Astragalus+Angelica sinensis(n=10) is also the combination with the lowest Level 3RR. RR of 13 TCM combinations was significantly higher than that of total pool (1.41). Level 4, combinations of 5 TMs, the most common combinations are Atractylodis macrocephala+Codonopsis+Astragalus+Poria cocos+Angelica sinensis (n=10; RR=1.95 [1.31, 2.90], I^2^ = 7), Atractylodis macrocephala+Poria cocos+Glycyrrhiza+Angelica sinensis +Curcuma zedoary (n=2) had the lowest RR value, 1.81 [1.00, 3.28]. The RR of the 7 TCM combinations was significantly higher than that of the total pool (1.41). **Table 5** shows that at Level 1, the most commonly used combination of macrocephala and Codonopsis was Atractylodis macrocephala (n=18; RR=1.86 [1.46, 2.37], I^2^ = 0), Atractylodis macrocephala+Poria cocos (n=18; RR=1.93 [1.52, 2.44], I^2^ = 0), the lowest RR of Atractylodis macrocephala+Malt (n=4) was 1.45 [0.89, 2.36], and the RR of 9 combined TCM combinations was significantly higher than that of the total pool (1.20). Level 2, combinations of 3 TMs, the most common combinations are: Atractylodis macrocephala+Codonopsis+Astragalus (n=13; RR=2.02 [1.51, 2.71], I^2^ = 7), Atractylodis macrocephala+Codonopsis+Poria cocos (n=13; RR=2.02 [1.51, 2.71], I^2^ = 7), Atractylodis macrocephala+Codonopsis+Angelica sinensis (n=13; RR=1.77 [1.34, 2.33], I^2^ = 0), Atractylodis macrocephala+Astragalus+Poria cocos (n=13; RR=2.34 [1.75, 3.12], I^2^ = 13), Atractylodis macrocephala+Poria cocos+Glycyrrhiza (n=13; RR=2.12 [1.61, 2.79], I^2^ = 0), the lowest RR of Atractylodis macrocephala+Panax ginseng+Astragalus (n=2) was 1.56 [0.77, 3.14]. RR of 19 TCM combinations was significantly higher than that of total pool (1.20).Level 3, combinations of 4 TMs, the most commonly used combination with the lowest RR is Atractylodis macrocephala+Codonopsis+Astragalus+Angelica sinensis (n=10; RR=2.12 [1.61, 2.79], I^2^ = 2), RR of 13 combinations was significantly higher than that of total pool RR (1.20).Level 4, combinations of 5 TMs, The most common combination is Atractylodis macrocephala+Codonopsis+Astragalus+Poria cocos+Angelica sinensis (n=7; RR=1.95 [1.31, 2.90], I^2^ = 7), RR of the 7 TCM combinations were significantly higher than that of the total pool (1.20). For Level 5 and Level 6, the combinations of Chinese medicines in **Table 4** and **Table 5** are the same. Atractylodis macrocephala+Codonopsis+Astragalus+Poria cocos+Glycyrrhiza+Angelica sinensis (n=4) and Atractylodis macrocephala+Codonopsis+Astragalus+Poria cocos+Glycyrrhiza+Angelica sinensis+Coix seed(n=2), RR values were greater than total pool RR.

**Table 4:** Effect of oral conventional drugs (TMs) on tumor response: Atractylodes macrocephala and combined TMs on ORR.

Level	TMPs	RR(95%CI)	N.stud.[Ref]	N.part.	I^2^
1	Atractylodis macrocephala+Codonopsis	1.86 [1.46, 2.37]	18 (13–19, 22, 24, 26–28, 32, 33, 37, 39–42)	1135	0
1	Atractylodis macrocephala+Astragalus	2.06 [1.59, 2.66]	17 (12–15, 18, 19, 24–26, 31, 32, 34, 37, 39–42)	1069	5
1	Atractylodis macrocephala+Poria cocos	1.98 [1.57, 2.49]	19 (13, 15, 17, 21, 22, 24–27, 29–31, 33, 34, 37, 39–42)	1307	0
1	Atractylodis macrocephala+Glycyrrhiza	1.97 [1.57, 2.48]	18 (17–19, 21, 24, 25, 27–34, 39–42)	1323	0
1	Atractylodis macrocephala+Angelica sinensis	1.94 [1.51, 2.49]	16 (13, 17–19, 21, 24, 26, 27, 30, 32–34, 37, 40–42)	1117	5
1	Atractylodis macrocephala+Curcuma zedoary	1.92 [1.42, 2.61]	11 (12, 14, 15, 18, 19, 27–29, 31, 32, 39)	714	0
1	Atractylodis macrocephala+Coix seed	1.74 [1.33, 2.28]	13 (12, 13, 22, 25–29, 31, 33, 37, 40, 42)	878	0
1	Atractylodis macrocephala+Malt	1.45 [0.89, 2.36]	4 (18, 22, 29, 42)	272	0
1	Atractylodis macrocephala+Germinatus Hordei Vulgaris	1.49 [0.85, 2.60]	3 (22, 29, 42)	212	0
1	Atractylodis macrocephala+Panax ginseng	2.36 [1.20, 4.63]	2 (25, 30)	152	0
2	Atractylodis macrocephala+Codonopsis+Astragalus	2.02 [1.51, 2.71]	13 (13–15, 18, 19, 24, 26, 32, 37, 39–42)	759	7
2	Atractylodis macrocephala+Codonopsis+Poria cocos	2.02 [1.51, 2.71]	13 (13–15, 18, 19, 24, 26, 32, 37, 39–42)	759	7
2	Atractylodis macrocephala+Codonopsis+Glycyrrhiza	1.85 [1.39, 2.45]	12 (17–19, 24, 27, 28, 32, 33, 39–42)	819	0
2	Atractylodis macrocephala+Codonopsis+Angelica sinensis	1.77 [1.34, 2.33]	13 (13, 17–19, 24, 26, 27, 32, 33, 37, 40–42)	851	0
2	Atractylodis macrocephala+Codonopsis+Curcuma zedoary	1.89 [1.37, 2.62]	9 (14, 15, 18, 19, 23, 27, 28, 32, 33, 39)	614	0
2	Atractylodis macrocephala+Astragalus+Poria cocos	2.34 [1.75, 3.12]	13 (13, 15, 21, 24–26, 31, 34, 37, 39–42)	847	13
2	Atractylodis macrocephala+Astragalus+Glycyrrhiza	2.16 [1.62, 2.89]	12 (18, 19, 21, 24, 25, 31, 32, 34, 39–42)	843	1
2	Atractylodis macrocephala+Astragalus+Angelica sinensis	1.91 [1.42, 2.57]	12 (13, 18, 19, 21, 24, 26, 32, 34, 37, 40–42)	797	22
2	Atractylodis macrocephala+Astragalus+Curcuma zedoary	2.18 [1.49, 3.19]	8 (12, 14, 15, 18, 19, 31, 32, 39)	474	0
2	Atractylodis macrocephala+Poria cocos+Glycyrrhiza	2.18 [1.68, 2.84]	14 (17, 21, 24, 25, 27, 29–31, 33, 34, 39–42)	1033	0
2	Atractylodis macrocephala+Poria cocos+Angelica sinensis	2.08 [1.57, 2.76]	13 (13, 17, 21, 24, 26, 27, 30, 33, 34, 37, 40–42)	907	6
2	Atractylodis macrocephala+Poria cocos+Curcuma zedoary	2.14 [1.47, 3.13]	6 (15, 27, 29, 31, 33, 39)	452	8
2	Atractylodis macrocephala+Glycyrrhiza+Angelica sinensis	2.00 [1.52, 2.64]	13 (17–19, 21, 24, 27, 30, 32–34, 40–42)	945	0
2	Atractylodis macrocephala+Glycyrrhiza+Curcuma zedoary	1.78 [1.31, 2.42]	9 (17, 19, 27–29, 31–33, 39)	696	0
2	Atractylodis macrocephala+Angelica sinensis+Curcuma zedoary	1.62 [1.08, 2.43]	5 (18, 19, 27, 32, 33)	390	0
2	Atractylodis macrocephala+Costusroot+Coix seed	1.81 [1.00, 3.28]	2 (27, 33)	180	0
2	Atractylodis macrocephala+Astragalus+Coix seed	2.00 [1.38, 2.90]	8 (12, 13, 16, 20, 25, 26, 31, 37, 38, 40, 42)	478	0
2	Atractylodis macrocephala+Poria cocos+Coix seed	1.80 [1.35, 2.40]	11 (13, 20, 22, 25–27, 29, 31, 33, 37, 38, 40, 42)	768	0
2	Atractylodis macrocephala+Panax ginseng+Astragalus	1.56 [0.77, 3.14]	2 (21, 25)	128	0
3	Atractylodis macrocephala+Astragalus+Poria cocos+Coix seed	2.03 [1.39, 2.97]	7 (13, 25, 26, 31, 37, 40, 42)	448	1
3	Atractylodis macrocephala+Codonopsis+Astragalus+Poria cocos	2.24 [1.57, 3.20]	9 (13, 15, 24, 26, 37, 39–42)	507	10
3	Atractylodis macrocephala+Codonopsis+Astragalus+Glycyrrhiza	1.99 [1.39, 2.84]	8 (18, 19, 24, 32, 39–42)	503	0
3	Atractylodis macrocephala+Codonopsis+Astragalus+Angelica sinensis	1.77 [1.29, 2.45]	10 (13, 18, 19, 24, 26, 32, 37, 40–42)	615	2
3	Atractylodis macrocephala+Codonopsis+Astragalus+Curcuma zedoary	2.11 [1.36, 3.27]	6 (14, 15, 18, 19, 32, 39)	354	22
3	Atractylodis macrocephala+Astragalus+Poria cocos+Glycyrrhiza	2.51 [1.78, 3.53]	9 (21, 24, 25, 31, 34, 39–42)	633	0
3	Atractylodis macrocephala+Astragalus+Poria cocos+Angelica sinensis	1.98 [1.40, 2.79]	9 (13, 21, 24, 26, 30, 37, 40–42)	549	0
3	Atractylodis macrocephala+Poria cocos+Glycyrrhiza+Angelica sinensis	2.22 [1.61, 3.06]	10 (17, 21, 24, 27, 30, 33, 34, 40–42)	735	0
3	Atractylodis macrocephala+Poria cocos+Glycyrrhiza+Curcuma zedoary	2.01 [1.35, 2.99]	5 (27, 29, 31, 33, 39)	410	7
3	Atractylodis macrocephala+Glycyrrhiza+Angelica sinensis +Curcuma zedoary	1.81 [1.17, 2.80]	4 (18, 27, 32, 33)	330	0
3	Atractylodis macrocephala+Codonopsis+Astragalus+Coix seed	2.49 [1.57, 3.96]	5 (13, 26, 37, 40, 42)	314	0
3	Atractylodis macrocephala+Astragalus+Poria cocos+Coix seed	2.03 [1.39, 2.97]	7 (13, 25, 26, 31, 37, 40, 42)	448	1
3	Atractylodis macrocephala+Poria cocos+Glycyrrhiza+Coix seed	1.91 [1.35, 2.71]	7 (25, 27, 29, 31, 33, 40, 42)	536	0
4	Atractylodis macrocephala+Codonopsis+Astragalus+Poria cocos+Coix seed	2.55 [1.49, 4.36]	5 (13, 26, 37, 40, 42)	234	0
4	Atractylodis macrocephala+Codonopsis+Astragalus+Poria cocos+Glycyrrhiza	2.48 [1.55, 3.99]	5 (24, 39–42)	293	0
4	Atractylodis macrocephala+Codonopsis+Astragalus+Poria cocos+Angelica sinensis	1.95 [1.31, 2.90]	7 (13, 24, 26, 37, 40–42)	405	7
4	Atractylodis macrocephala+Codonopsis+Astragalus+Poria cocos+Curcuma zedoary	4.08 [1.77, 9.40]	2 (15, 39)	102	0
4	Atractylodis macrocephala+Astragalus+Poria cocos+Glycyrrhiza+Angelica sinensis	2.42 [1.56, 3.75]	6 (21, 24, 34, 40–42)	415	12
4	Atractylodis macrocephala+Astragalus+Poria cocos+Glycyrrhiza+Curcuma zedoary	3.18 [1.63, 6.23]	2 (31, 39)	150	0
4	Atractylodis macrocephala+Poria cocos+Glycyrrhiza+Angelica sinensis +Curcuma zedoary	1.81 [1.00, 3.28]	2 (27, 33)	180	0
5	Atractylodis macrocephala+Codonopsis+Astragalus+Poria cocos+Glycyrrhiza+Angelica sinensis	2.22 [1.30, 3.76]	4 (24, 40–42)	233	0
6	Atractylodis macrocephala+Codonopsis+Astragalus+Poria cocos+Glycyrrhiza+Angelica sinensis+Coix seed	2.38 [1.11, 5.06]	2 (40, 42)	118	0

Conclusion: In TM intervention, Atractylodes macrocephala combined with other drugs has a strong synergistic effect on ORR.

**Table 5:** Effect of oral conventional drugs (TMs) on tumor response:Atractylodes macrocephala and combined TMs on DCR.

Level	TMPs	RR(95%CI)	N.stud.[Ref]	N.part.	I^2^
1	Atractylodis macrocephala+Codonopsis	1.86 [1.46, 2.37]	18 (13–19, 22, 24, 26–28, 32, 33, 37, 39–42)	1135	0
1	Atractylodis macrocephala+Astragalus	2.06 [1.59, 2.66]	17 (12–15, 18, 19, 24–26, 31, 32, 34, 37, 39–42)	1069	5
1	Atractylodis macrocephala+Poria cocos	1.93 [1.52, 2.44]	18 (13, 15, 17, 21, 22, 24–27, 29, 31, 33, 34, 37, 39–42)	1223	0
1	Atractylodis macrocephala+Glycyrrhiza	1.92 [1.52, 2.43]	17 (17–19, 21, 24, 25, 27–29, 31–34, 39–42)	1239	0
1	Atractylodis macrocephala+Angelica sinensis	1.88 [1.45, 2.43]	15 (13, 17–19, 21, 24, 26, 27, 32–34, 37, 40–42)	1033	5
1	Atractylodis macrocephala+Curcuma zedoary	1.92 [1.42, 2.61]	11 (12, 14, 15, 18, 19, 27–29, 31, 32, 39)	714	0
1	Atractylodis macrocephala+Coix seed	1.74 [1.33, 2.28]	13 (12, 13, 22, 25–29, 31, 33, 37, 40, 42)	878	0
1	Atractylodis macrocephala+Malt	1.45 [0.89, 2.36]	4 (18, 22, 29, 42)	272	0
1	Atractylodis macrocephala+Germinatus Hordei Vulgaris	1.49 [0.85, 2.60]	3 (22, 29, 42)	212	0
2	Atractylodis macrocephala+Codonopsis+Astragalus	2.02 [1.51, 2.71]	13 (13–15, 18, 19, 24, 26, 32, 37, 39–42)	759	7
2	Atractylodis macrocephala+Codonopsis+Poria cocos	2.02 [1.51, 2.71]	13 (13–15, 18, 19, 24, 26, 32, 37, 39–42)	759	7
2	Atractylodis macrocephala+Codonopsis+Glycyrrhiza	1.85 [1.39, 2.45]	12 (17–19, 24, 27, 28, 32, 33, 39–42)	819	0
2	Atractylodis macrocephala+Codonopsis+Angelica sinensis	1.77 [1.34, 2.33]	13 (13, 17–19, 24, 26, 27, 32, 33, 37, 40–42)	851	0
2	Atractylodis macrocephala+Codonopsis+Curcuma zedoary	1.89 [1.37, 2.62]	9 (14, 15, 18, 19, 23, 27, 28, 32, 33, 39)	614	0
2	Atractylodis macrocephala+Astragalus+Poria cocos	2.34 [1.75, 3.12]	13 (13, 15, 21, 24–26, 31, 34, 37, 39–42)	847	13
2	Atractylodis macrocephala+Astragalus+Glycyrrhiza	2.16 [1.62, 2.89]	12 (18, 19, 21, 24, 25, 31, 32, 34, 39–42)	843	1
2	Atractylodis macrocephala+Astragalus+Angelica sinensis	1.91 [1.42, 2.57]	12 (13, 18, 19, 21, 24, 26, 32, 34, 37, 40–42)	797	22
2	Atractylodis macrocephala+Astragalus+Curcuma zedoary	2.18 [1.49, 3.19]	8 (12, 14, 15, 18, 19, 31, 32, 39)	474	0
2	Atractylodis macrocephala+Poria cocos+Glycyrrhiza	2.12 [1.61, 2.79]	13 (17, 21, 24, 25, 27, 29, 31, 33, 34, 39–42)	949	0
2	Atractylodis macrocephala+Poria cocos+Angelica sinensis	2.01 [1.49, 2.70]	12 (13, 17, 21, 24, 26, 27, 33, 34, 37, 40–42)	823	8
2	Atractylodis macrocephala+Poria cocos+Curcuma zedoary	2.14 [1.47, 3.13]	6 (15, 27, 29, 31, 33, 39)	452	8
2	Atractylodis macrocephala+Glycyrrhiza+Angelica sinensis	1.93 [1.45, 2.57]	12 (17–19, 21, 24, 27, 32–34, 40–42)	861	0
2	Atractylodis macrocephala+Glycyrrhiza+Curcuma zedoary	1.78 [1.31, 2.42]	9 (17, 19, 27–29, 31–33, 39)	696	0
2	Atractylodis macrocephala+Angelica sinensis+Curcuma zedoary	1.62 [1.08, 2.43]	5 (18, 19, 27, 32, 33)	390	0
2	Atractylodis macrocephala+Costusroot+Coix seed	1.81 [1.00, 3.28]	2 (27, 33)	180	0
2	Atractylodis macrocephala+Astragalus+Coix seed	2.00 [1.38, 2.90]	8 (12, 13, 16, 20, 25, 26, 31, 37, 38, 40, 42)	478	0
2	Atractylodis macrocephala+Poria cocos+Coix seed	1.80 [1.35, 2.40]	11 (13, 20, 22, 25–27, 29, 31, 33, 37, 38, 40, 42)	768	0
2	Atractylodis macrocephala+Panax ginseng+Astragalus	1.56 [0.77, 3.14]	2 (21, 25)	128	0
3	Atractylodis macrocephala+Astragalus+Poria cocos+Coix seed	2.03 [1.39, 2.97]	7 (13, 25, 26, 31, 37, 40, 42)	448	1
3	Atractylodis macrocephala+Codonopsis+Astragalus+Poria cocos	2.24 [1.57, 3.20]	9 (13, 15, 24, 26, 37, 39–42)	507	10
3	Atractylodis macrocephala+Codonopsis+Astragalus+Glycyrrhiza	1.99 [1.39, 2.84]	8 (18, 19, 24, 32, 39–42)	503	0
3	Atractylodis macrocephala+Codonopsis+Astragalus+Angelica sinensis	1.77 [1.29, 2.45]	10 (13, 18, 19, 24, 26, 32, 37, 40–42)	615	2
3	Atractylodis macrocephala+Codonopsis+Astragalus+Curcuma zedoary	2.11 [1.36, 3.27]	6 (14, 15, 18, 19, 32, 39)	354	22
3	Atractylodis macrocephala+Astragalus+Poria cocos+Glycyrrhiza	2.51 [1.78, 3.53]	9 (21, 24, 25, 31, 34, 39–42)	633	0
3	Atractylodis macrocephala+Astragalus+Poria cocos+Angelica sinensis	1.85 [1.28, 2.68]	8 (13, 21, 24, 26, 37, 40–42)	465	0
3	Atractylodis macrocephala+Poria cocos+Glycyrrhiza+Angelica sinensis	2.13 [1.52, 3.00]	9 (17, 21, 24, 27, 33, 34, 40–42)	651	0
3	Atractylodis macrocephala+Poria cocos+Glycyrrhiza+Curcuma zedoary	2.01 [1.35, 2.99]	5 (27, 29, 31, 33, 39)	410	7
3	Atractylodis macrocephala+Glycyrrhiza+Angelica sinensis +Curcuma zedoary	1.81 [1.17, 2.80]	4 (18, 27, 32, 33)	330	0
3	Atractylodis macrocephala+Codonopsis+Astragalus+Coix seed	2.49 [1.57, 3.96]	5 (13, 26, 37, 40, 42)	314	0
3	Atractylodis macrocephala+Astragalus+Poria cocos+Coix seed	2.03 [1.39, 2.97]	7 (13, 25, 26, 31, 37, 40, 42)	448	1
3	Atractylodis macrocephala+Poria cocos+Glycyrrhiza+Coix seed	1.91 [1.35, 2.71]	7 (25, 27, 29, 31, 33, 40, 42)	536	0
4	Atractylodis macrocephala+Codonopsis+Astragalus+Poria cocos+Coix seed	2.55 [1.49, 4.36]	5 (13, 26, 37, 40, 42)	234	0
4	Atractylodis macrocephala+Codonopsis+Astragalus+Poria cocos+Glycyrrhiza	2.48 [1.55, 3.99]	5 (24, 39–42)	293	0
4	Atractylodis macrocephala+Codonopsis+Astragalus+Poria cocos+Angelica sinensis	1.95 [1.31, 2.90]	7 (13, 24, 26, 37, 40–42)	405	7
4	Atractylodis macrocephala+Codonopsis+Astragalus+Poria cocos+Curcuma zedoary	4.08 [1.77, 9.40]	2 (15, 39)	102	0
4	Atractylodis macrocephala+Astragalus+Poria cocos+Glycyrrhiza+Angelica sinensis	2.42 [1.56, 3.75]	6 (21, 24, 34, 40–42)	415	12
4	Atractylodis macrocephala+Astragalus+Poria cocos+Glycyrrhiza+Curcuma zedoary	3.18 [1.63, 6.23]	2 (31, 39)	150	0
4	Atractylodis macrocephala+Poria cocos+Glycyrrhiza+Angelica sinensis +Curcuma zedoary	1.81 [1.00, 3.28]	2 (27, 33)	180	0
5	Atractylodis macrocephala+Codonopsis+Astragalus+Poria cocos+Glycyrrhiza+Angelica sinensis	2.22 [1.30, 3.76]	4 (24, 40–42)	233	0
6	Atractylodis macrocephala+Codonopsis+Astragalus+Poria cocos+Glycyrrhiza+Angelica sinensis+Coix seed	2.38 [1.11, 5.06]	2 (40, 42)	118	0

Conclusion: In TM intervention, Atractylodes macrocephala combined with other drugs has a strong synergistic effect on DCR.

### 3.9 Sensitivity analysis

We conducted analyses to evaluate the robustness by systematically excluding each investigation to evaluate the reliability pertaining to the primary outcomes, which included ORR, DCR, QOL, and ADRs. The ORR, and the combined RR values of the ADRs were stable (**Table 6**). Considering the DCR dichotomous variable extracted from Zhu et al., 2019 (40), which this study does not include, I^2^ declined from 62% to 0%. Considering the QOL continuous class variable data extracted from Li et al., 2019 (34), Chen et al., 2013 (49), we excluded these 2 studies and I^2^ decreased from 80% to 0%.

**Table 6:** Egger’s test.

Meta-analysis of publication bias	P value
DCR	0.015
QOL	0.035
CD3^+^T cells	0.235
CD4^+^T cells	0.34
CD8^+^T cells	0.632
CD4^+^CD8^+^T cells	0.704

DCR, disease control rate; QOL, quality of life.

Conclusions: Although DCR, QOL, CD3+T cells, CD4+T cells, CD8+T cells, CD4+CD8+T cells I^2^ were large, there was no significant publication bias in the analysis results.

### 3.10 Publication bias

The funnel plots showed some asymmetry and were not perfectly symmetrical in the meta-analysis of DCR, QOL, CD3^+^T cells, CD4^+^T cells, CD8^+^T cells, and CD4^+^CD8^+^T cells ratios(S1-S6). However, Egger’s test (**Table 6**) showed that there was no significant publication bias in the meta-analysis of CD3^+^T cells (P= 0.235), CD4^+^T cells (P=0.34), CD8^+^T cells (P= 0.632), and CD4^+^CD8^+^T cells (P= 0.704.) The meta-analysis of DCR (P= 0.015) (P= 0.015), and QOL (P= 0.035) meta-analysis all had significant publication bias.

### 3.11 Quality of evidence

In conclusion, regarding ORR, QOL dichotomous variables, as well as thrombocytopenia, nausea and vomiting, diarrhea, and hepatorenal dysfunction, CD3+T Cells, CD4+T Cells, CD8+T Cells, CD4+CD8+T Cells, the quality was moderate; DCR, Neutropenia, Anemia, Skin Toxicity, Stomatitis, Neurotoxicity, Hand-Foot Syndrome, Alopecia, and KPS, according to mean ± SD had very low results (**Table 7**).

**Table 7:** GRADE evidence profile.

Outcomes (Trials)	Quality assessment					No. of patients		Risk ratios (95% CI)	Quality
	Risk of bias	Inconsistency	Indirectness	Imprecision	Reporting bias	Atractylodis-Containing TCM	NAC		
ORR(25)	Serious^a^	NO	NO	NO	NO	435/834(52.2%)	307/835(36.8%)	RR 1.41(1.27 to 1.57)	⊕⊕⊕O Moderate
DCR(24)	very serious^b^	NO	NO	NO	NO	649/792(81.9%)	544/795(68.4%)	RR 1.20(1.13 to 1.27)	⊕⊕OO Low
KPS(13)	Serious^a^	NO	NO	NO	NO	348/420(82.9%)	241/417(57.8%)	RR 1.43(1.30 to 1.57)	⊕⊕⊕O Moderate
Neutropenia(10)	Serious^a^	NO	NO	NO	NO	56/342(16.4%)	93/338(27.5%)	RR 0.47 (0.31 to 0.70)	⊕⊕⊕O Moderate
Anemia(3)	Serious^a^	NO	NO	NO	Serious^e^	20/107(18.7%)	36/103(35.0%)	RR 0.43 (0.23 to 0.81)	⊕⊕OO Low
Thrombocytopenia(4)	Serious^a^	NO	NO	NO	Serious^e^	21/137(15.3%)	40/133(30.1%)	RR 0.41 (0.22 to 0.75)	⊕⊕OO Low
Nausea and vomiting(10)	Serious^a^	NO	NO	NO	NO	96/331(29%)	163/325(50.2%)	RR 0.33 (0.23 to 0.48)	⊕⊕⊕O Moderate
Diarrhea(7)	Serious^a^	NO	NO	NO	Serious^e^	40/233(17.2%)	75/229(32.8%)	RR 0.36 (0.22 to 0.58)	⊕⊕OO Low
Hepatorenal dysfunction(5)	Serious^a^	NO	NO	NO	Serious^e^	10/180(5.6%)	27/180(15%)	RR 0.33 (0.15 to 0.70)	⊕⊕OO Low
CD3+T cells (5)	Serious^a^	NO^1^	NO	NO	NO	186	186	MD 9.44 higher (8.43 to 10.45 higher)	⊕⊕⊕O Moderate
CD4+T cells (6)	Serious^a^	NO^1^	NO	NO	NO	207	207	MD 3.90 higher (3.16 to 4.64 higher)	⊕⊕⊕O Moderate
CD8+T cells (5)	Serious^a^	NO^1^	NO	NO	NO	162	162	MD -0.55 higher (-1.39 to 0.29 higher)	⊕⊕⊕O Moderate
CD4+/CD8+T cells (6)	Serious^a^	NO^1^	NO	NO	NO	207	207	MD 0.13 higher (0.08 to 0.19 higher)	⊕⊕⊕O Moderate
KPS, according to mean ± SD	Serious^a^	NO	NO	NO	Serious^e^	180	147	MD 8.47 higher (7.16 to 9.77 higher)	⊕⊕OO Low

ORR, objective response rate; DCR, disease control rate; QOL, quality of life; CI, confidence interval.

a Most trials had unclear risk, and with high risk, but the result had good robustness. The evidence was rated down by only one level.

b Most trials had unclear risk, and with high risk, and the result had poor robustness. The evidence was rated down by two level.

c Heterogeneity presented in them, and the results had good robustness. Not rated down.

d Heterogeneity presented in them, and the results had poor robustness. The evidence was rated down by one level.

e The sample size for each outcome was fewer than 300 cases. Therefore, the evidence was rated down by one level.

Conclusion: The GRADE evidence summary classifies the quality of each piece of evidence as high, medium, low or very low to indicate the degree of certainty of the conclusion. The evidence quality in this study is relatively reliable.

## 4 Discussion

Cancer is not only a worldwide health problem, but it also poses a serious danger to both individuals and society, serving as a major threat to public health. Cancer is the primary reason for most deaths in every country around the world, with millions of individuals losing their lives to cancer every year, and a huge obstacle in increasing life expectancy of mankind (52). According to the U.S. Cancer Report 2023, gastric cancer occupies the top 6 places in terms of incidence and mortality of highly prevalent malignant tumors in various countries and regions around the world (53). In China, in 2022, the number of incidence cases of malignant tumors in China was about 4,824,700 cases, and the number of death cases was about 2,574,200 cases, and gastric cancer ranked 5th among the cancer types with the number of death cases of malignant tumors in China (54). Early gastric cancer patients often develop AGC because of nonspecific symptoms such as belching and epigastric discomfort, or even no obvious symptoms. neoadjuvant chemotherapy is a short-term chemotherapy intervention before surgical treatment, which can promote tumor shrinkage and improve the effect of surgical treatment (55). Nevertheless, the survival advantage for patients treatment of patients with advanced gastric cancer remains constrained. Therefore, there is a need for developing an effective portfolio program therapy regimen to improve the effectiveness of NAC, reducing adverse impacts, and improve patient compliance and elapsed time for survival, thereby enhancing the quality of patients’ lives.

Chinese medicine compounding, being a crucial component of a comprehensive gastric cancer treatment, is commonly employed in gastric cancer patients in China (56).

In order to assess whether oral Atractylodes macrocephala-containing Chinese herbal preparations combined with NAC could improve the clinical efficacy and safety, 32 randomized controlled trials involving 2157 AGC practitioner participated in this meta-analysis. Our findings demonstrated that the herbal combination containing Atractylodes enhanced the ORR and DCR of NAC, suggesting that the intervention group exhibited superior short-term effectiveness. Additionally, we conducted subgroup analyses according to Atractylodes macrocephala-containing herbal treatment procedures, NAC regimens, and length of therapy. The results showed that ORR and DCR could be improved in AGC patients with or without initial treatment. Oral administration of Atractylodes macrocephala-containing herbs had better tumor efficacy in patients with AGC compared to NAC alone. A chemotherapy regimen in which Atractylodes macrocephala-containing herbs were given in combination with fluorouracil-based chemotherapy and oxaliplatin in patients with AGC improved the ORR and the DCR; however, DCR did not improve in those who took aloe-containing herbs plus cisplatin. The subgroup analysis results also showed that the ORR and DCR could be improved by Atractylodes macrocephala-containing herbs plus NAC in patients with AGC, regardless of the duration of administration. Additionally, researchers have isolated a variety of chemicals from Atractylodes macrocephala. Among them, atractylodes macrocephalae polysaccharides consist of various groups of sugars, including arabinose (Ara), glucose (Glc), mannose (Man), rhamnose (Rha), galacturonic acid (GalA), galactose (Gal), and xylose (Xyl), among others (57).Its anticancer activity is mediated in several ways: modulation of the tumor microenvironment and immune function, inhibition of tumor cell migration and invasion and metastasis, induction of apoptosis and inhibition of tumor cell proliferation (58), and it has a wide range of antitumor activity against a variety of tumor cell types like esophageal cancer, hepatocellular carcinoma, malignant glioma, colorectal cancer, lung cancer cells, etc (59, 60). The volatile oil of Atractylodes macrocephala exemplifies good antitumor effects through its suppressing effects on a range of tumor cells (61). Both Atractylenolide I and Atractylenolide II have antitumor effects. In addition to the antitumor effects of atractylenolide I on lung cancer cells, ovarian cancer cells, and leukemia cells *in vitro* experiments (62–64), *in vivo* the results of the study show that Atractylenolide 1 significantly inhibited the tumor body of SGC-7901 gastric cancer cells in BALB/C nude mice with loaded mice by increasing the expression of Bax, cleaved caspase-3, and p53, and decreasing the manifestation of Bcl-2 proliferation (65). Atractylenolide II significantly induces apoptosis in HGC-27 and AGS gastric cancer cells by deactivating Pathways of the Ras/ERK and PI3K/AKT signaling pathways (66, 67). These findings provide indirect evidence for the basis and principle of the anticancer mechanism of AGC containing Atractylodes macrocephala components.

NAC can lead to multiple adverse effects in AGC patients, which can seriously affect quality of life and survival expectations. Therefore, it is essential to maintain efficacy while minimizing adverse effects. In one study, AM(Atractylodes macrocephala) extract significantly promotes the migration of intestinal epithelial cells by modulating the polyamine Kv1 signaling pathway.1.This suggests that atractylodes helps promote the repair of intestinal damage (68). AM polysaccharides are beneficial to functions of the digestive tract, as the presence of chitin is associated with recovery from intestinal damage (69). In a rat experimental model, atractylenolide I and atractylenolide III significantly reduced muscle contraction in isolated ileum (70). Atractylenolide I improves the malignant quality of tumor patients, and atractylenolide I treatment increases body weight, appetite, KPS score, and TNF-α level in gastric cancer patients (71, 72). In serum-deficiency-induced PC12 cells, atractylenolide III inhibited apoptosis by increasing cellular activity, suggesting that Atractylenolide III has significant neuroprotective effects (73).

According to our findings, Atractylodis Macrocephalae-containing herbs combined with NAC reduced ADRs in AGC patients (Neutropenia, Anemia, Thrombocytopenia, Nausea and vomiting, Diarrhea, Hepatic and Renal dysfunction, Stomatitis, Neurotoxicity, Skin Toxicity, Hand-Foot Syndrome, Alopecia) (P < 0.05) and significantly improved KPS-based QOL.

T lymphocytes are important immunoregulatory cells and effector cells of human cellular immunity, which play a significant role in inhibiting the proliferation of tumor cells and maintaining the immune function of the human body, therefore, T lymphocyte subpopulations can be used to assess the cellular immune system function the organism. Some studies have pointed out that the function of the human immune system can partly reflect the prognosis and development of gastric cancer (74).

Therefore, improving immunity is important for human health anti-tumor therapy. An animal study showed that AM polysaccharides increased peripheral blood lymphoid levels and their conversion rate, as well as the expression of IL-6, TNF-α, IFN-γ, and nuclear factor-activated transcription factor of activated T-cells (NF-AT) in splenic tissues of piglets. These effects suggest that AM polysaccharides may enhance immune responses by promoting lymphocyte proliferation (75). A study using a chicken spleen tissue model system found that HS increased the expression of pro-apoptosis-related genes such as IL-1 and TNF-α, and decreased the expression of mitochondrial and anti-apoptosis-related genes such as IL-2 and IFN-γ. Studies have shown that AM polysaccharides mitigate these adverse effects by reducing oxidative stress, enhancing mitochondrial function, and inhibiting apoptosis; this finding suggests that AM polysaccharides may play a role in protecting immune organs (76). In a mouse airbag model system, extracts from AM inhibited the production of NO, TNF-α, IL-1β, IL-6, VEGF and PlGF. This suggests a strong inhibitory effect of Atractylodes macrocephala in the treatment of chronic inflammation (77). In RAW264.7 cells, the addition of AM extracts resulted in a dose-dependent decrease in NO production after LPS stimulation. This suggests that AM extracts have the same anti-inflammatory activity (78).

There are several limitations to this study. Firstly, we only searched for literature in Chinese and English databases, which may have caused us to miss some relevant studies in Japanese, Korean, or other language databases. All randomized controlled trials in our research was conducted in China, and the results from the funnel plot and Egger’s test suggest that there may be possible publication bias. In addition, only eight studies explicitly described the method of randomized allocation. In most of the included trials, hiding the mandate and blindness were labeled as “unclear,” which could lead to possible implementation bias and selectivity bias. Third, all of the randomized controlled trials in our study did not have tumor markers as a key indicator; therefore, we look forward to conducting more studies on tumor markers containing Atractylodes macrocephala. Fourth, many outcomes, such as DCR, QOL, and peripheral blood lymphocyte levels, showed significant differences (I^2^>50%) between studies, which may affect the reliability of the meta-analysis results. Therefore, it is necessary to conduct more rigorously designed randomized controlled trials to validate our findings. Fifth, Atractylodes macrocephala is rarely used as a monotherapy in the treatment of AGC; it is usually used in combination with other different herbs. The mechanism of specific immunity and cytotoxicity of white atractylodes in adjuvant chemotherapy for AGC needs to be further investigated. Sixth, in all the included RCTs, only oral treatments containing Atractylodes macrocephala herbal medicine were available; injections and other preparations were not found; more preparations containing Atractylodes macrocephala herbal medicine need to be further investigated. Finally, based on the GRADE approach, the quality of the results ranged from moderate to exceedingly low; most of the trials incorporated in the original manuscript may not have been reported strictly according to the CONSORT reporting criteria. All these limitations may have led to an inadequate assessment of the results. However, we aspire that our manuscript will encourage an increasing number of physicians to acknowledge the potential advantages of herbal medicines containing white herbs in AGC. We also hope to inspire the undertaking of more meticulously planned clinical trials in the future to validate the effectiveness of herbal medicines containing white herbs in alignment with the CONSORT guidelines.

## 5 Conclusion

Our study showed that the use of herbal medicine containing Atractylodis Macrocephalae in combination with NAC had improved outcomes and safety for patients with gastric adenocarcinoma. Additional endeavors are required to advocate for the utilization of Atractylodis Macrocephalae-containing herbal medicines in clinical environments.

Furthermore, the lasting effectiveness of Atractylodis Macrocephalae-containing herbal remedies in conjunction with NAC for treating AGC should be confirmed in upcoming meticulously planned clinical studies that adhere to the CONSORT guidelines.

## Data Availability

The original contributions presented in the study are included in the article/**Supplementary Material**. Further inquiries can be directed to the corresponding authors.
