# Supplementary material for: Efficacy and safety of Atractylodes macrocephala-containing traditional Chinese medicine combined with neoadjuvant chemotherapy in the treatment of advanced gastric cancer: a systematic evaluation and meta-analysis
Source: Front Oncol. 2024 Oct 16;14:1431381. doi: 10.3389/fonc.2024.1431381 (PMC11521787; doi:10.3389/fonc.2024.1431381)
Supplement: Supplementary file 1 [file DataSheet1.pdf]

# Supplementary Material

## 1 Supplementary Data

#1 (((((((((((((((((((stomach neoplasms[Title/Abstract]) OR (gastric cancer[Title/Abstract])) OR (Neoplasm, Stomach[Title/Abstract])) OR (Stomach Neoplasm[Title/Abstract])) OR (Neoplasms, Stomach[Title/Abstract])) OR (Gastric Neoplasms[Title/Abstract])) OR (Gastric Neoplasm[Title/Abstract])) OR (Neoplasm, Gastric[Title/Abstract])) OR (Neoplasms, Gastric[Title/Abstract])) OR (Cancer of Stomach[Title/Abstract])) OR (Stomach Cancers[Title/Abstract])) OR (Gastric Cancer[Title/Abstract])) OR (Cancer, Gastric[Title/Abstract])) OR (Cancers, Gastric[Title/Abstract])) OR (Gastric Cancers[Title/Abstract])) OR (Stomach Cancer[Title/Abstract])) OR (Cancer, Stomach[Title/Abstract])) OR (Cancers, Stomach[Title/Abstract])) OR (Cancer of the Stomach[Title/Abstract])) OR (Gastric Cancer, Familial Diffuse[Title/Abstract])

#2 ((((((((((((((((((((((((("Atractylodes"[Mesh]) OR (Jutsu[Title/Abstract])) OR (Jutsus[Title/Abstract])) OR (Atractylodes lancea[Title/Abstract])) OR (Atractylodes lanceas[Title/Abstract])) OR (lanceas, Atractylodes[Title/Abstract])) OR (Atractylenolide[Title/Abstract])) OR (β-eudesmol[Title/Abstract])) OR (Atractylodes macrocephala polysaccharides[Title/Abstract])) OR (Atractylodes rhizome[Title/Abstract])) OR (Atractylodin[Title/Abstract])) OR (Yupingfeng san[Title/Abstract])) OR (Guiqibaizhu[Title/Abstract])) OR (Fupihualiu[Title/Abstract])) OR (Jianpiyishen[Title/Abstract])) OR (Guipi[Title/Abstract])) OR (Sijunzi[Title/Abstract])) OR (Jianpiyangwei[Title/Abstract])) OR (Bazhentang[Title/Abstract])) OR (Huangqisijunzi[Title/Abstract])) OR (Buqiyangxue[Title/Abstract])) OR (Jianpifuzheng[Title/Abstract])) OR (Fuzhengquxiejiedu[Title/Abstract])) OR (Buzhonyiqi[Title/Abstract])) OR (Yiqihuayujiedu[Title/Abstract])

#3 ((((((((((((((((((((stomach neoplasms[Title/Abstract]) OR (gastric cancer[Title/Abstract])) OR (Neoplasm, Stomach[Title/Abstract])) OR (Stomach Neoplasm[Title/Abstract])) OR (Neoplasms, Stomach[Title/Abstract])) OR (Gastric Neoplasms[Title/Abstract])) OR (Gastric Neoplasm[Title/Abstract])) OR (Neoplasm, Gastric[Title/Abstract])) OR (Neoplasms, Gastric[Title/Abstract])) OR (Cancer of Stomach[Title/Abstract])) OR (Stomach Cancers[Title/Abstract])) OR (Gastric Cancer[Title/Abstract])) OR (Cancer, Gastric[Title/Abstract])) OR (Cancers, Gastric[Title/Abstract])) OR (Gastric Cancers[Title/Abstract])) OR (Stomach Cancer[Title/Abstract])) OR (Cancer, Stomach[Title/Abstract])) OR (Cancers, Stomach[Title/Abstract])) OR (Cancer of the Stomach[Title/Abstract])) OR (Gastric Cancer, Familial Diffuse[Title/Abstract])) AND (((((((((((((((((((((((((("Atractylodes"[Mesh]) OR (Jutsu[Title/Abstract])) OR (Jutsus[Title/Abstract])) OR (Atractylodes lancea[Title/Abstract])) OR (Atractylodes lanceas[Title/Abstract])) OR (lanceas, Atractylodes[Title/Abstract])) OR (Atractylenolide[Title/Abstract])) OR (β-eudesmol[Title/Abstract])) OR (Atractylodes macrocephala polysaccharides[Title/Abstract])) OR (Atractylodes rhizome[Title/Abstract])) OR (Atractylodin[Title/Abstract])) OR (Yupingfeng san[Title/Abstract])) OR (Guiqibaizhu[Title/Abstract])) OR (Fupihualiu[Title/Abstract])) OR (Jianpiyishen[Title/Abstract])) OR (Guipi[Title/Abstract])) OR (Sijunzi[Title/Abstract])) OR (Jianpiyangwei[Title/Abstract])) OR (Bazhentang[Title/Abstract])) OR

(Huangqisijunzi[Title/Abstract])) OR (Buqiyangxue[Title/Abstract])) OR (Jianpifuzheng[Title/Abstract])) OR (Fuzhengquxiejiedu[Title/Abstract])) OR (Buzhonyiqi[Title/Abstract])) OR (Yiqihuayujiedu[Title/Abstract]))

# 2 Supplementary Figures and Tables

## 2.1 Supplementary Figures

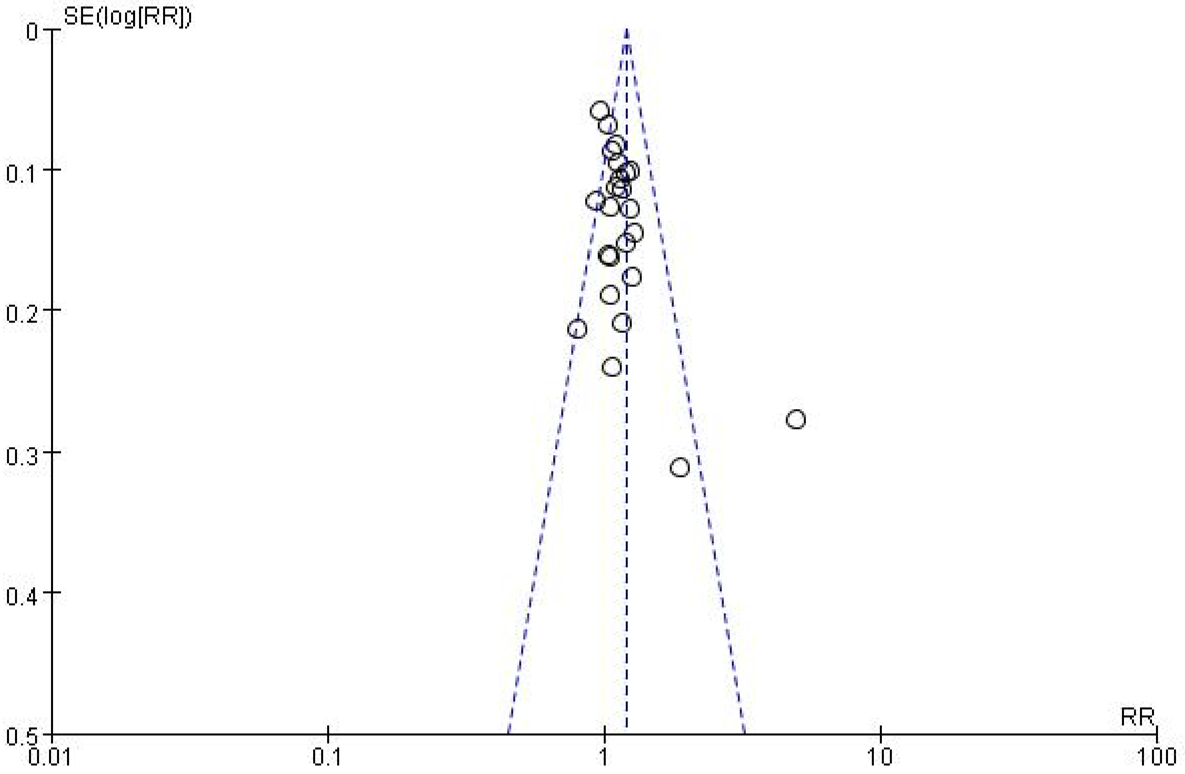


**Figure S1. DCR funnel plot**

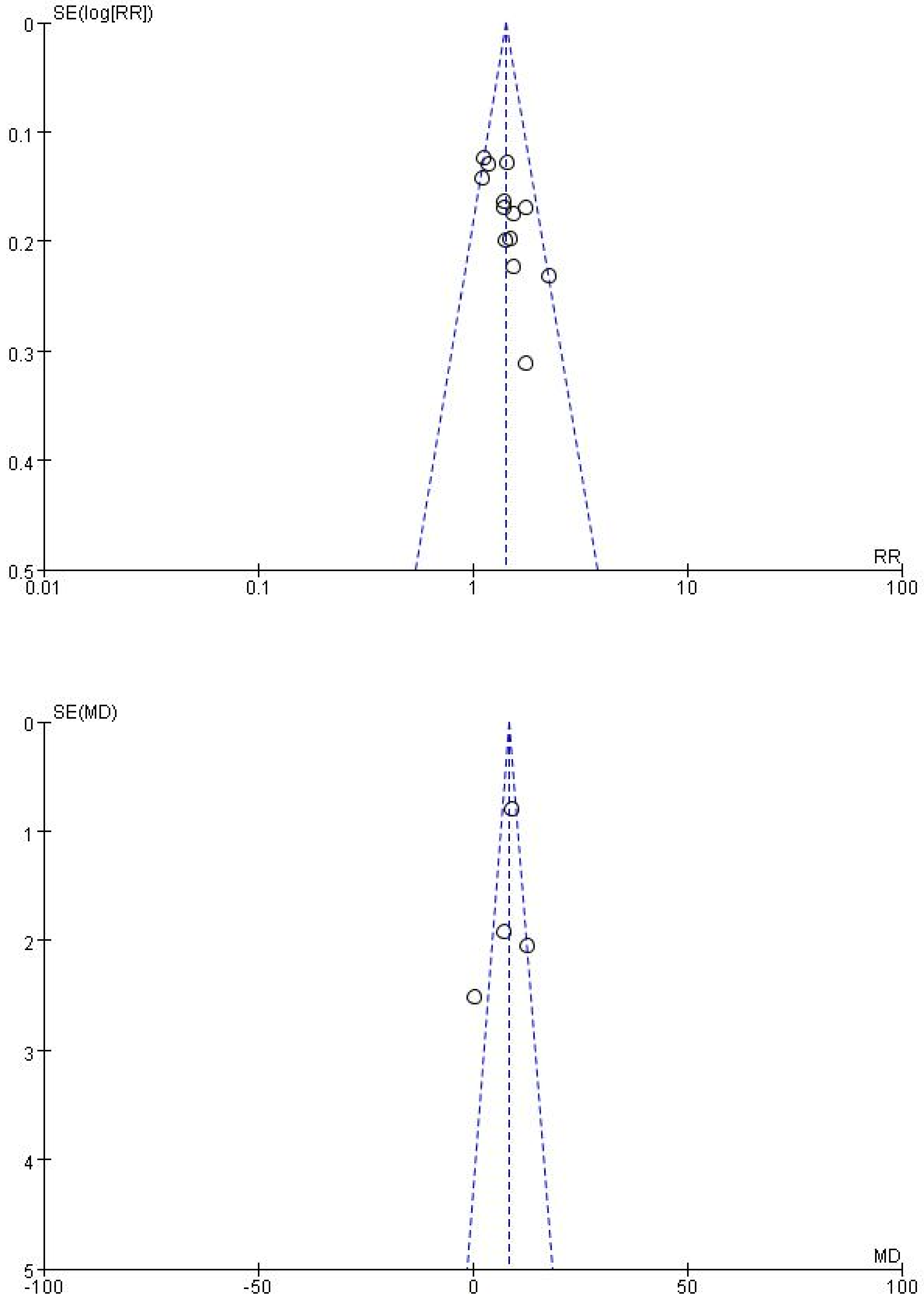


**Figure S2. QOL funnel plot**

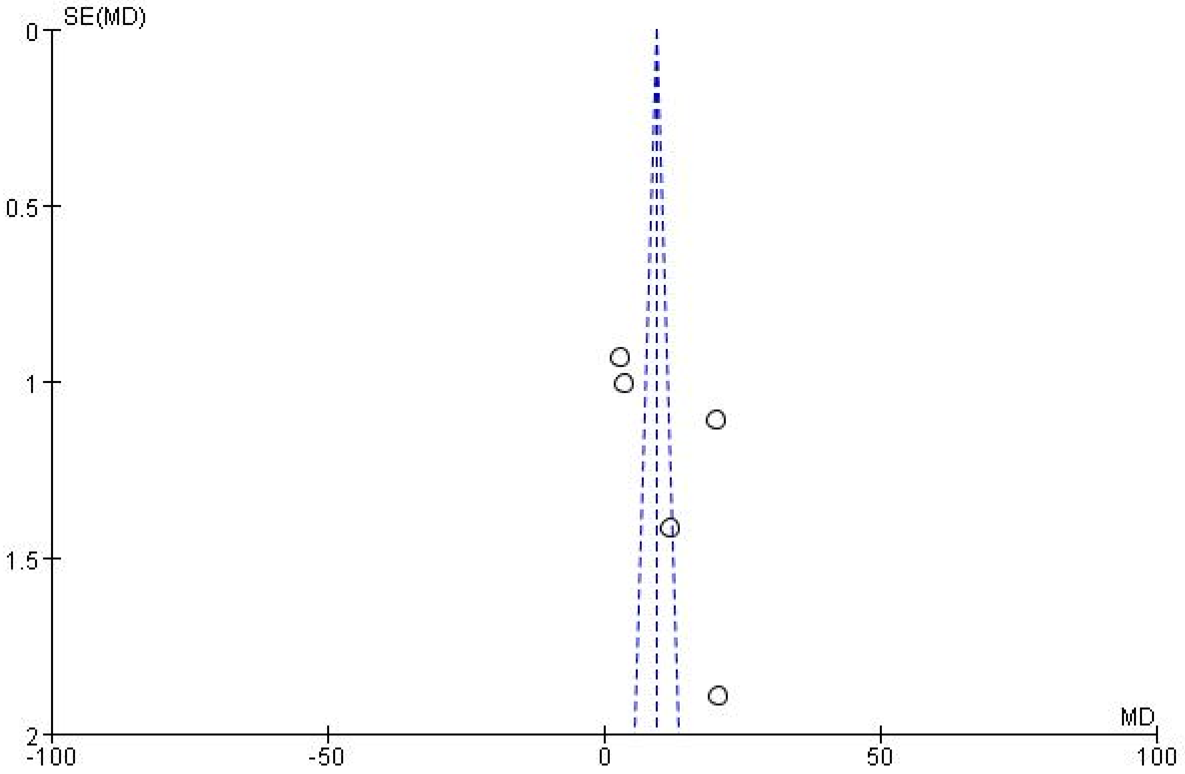


**Figure S3. CD3+ T cells funnel plot**

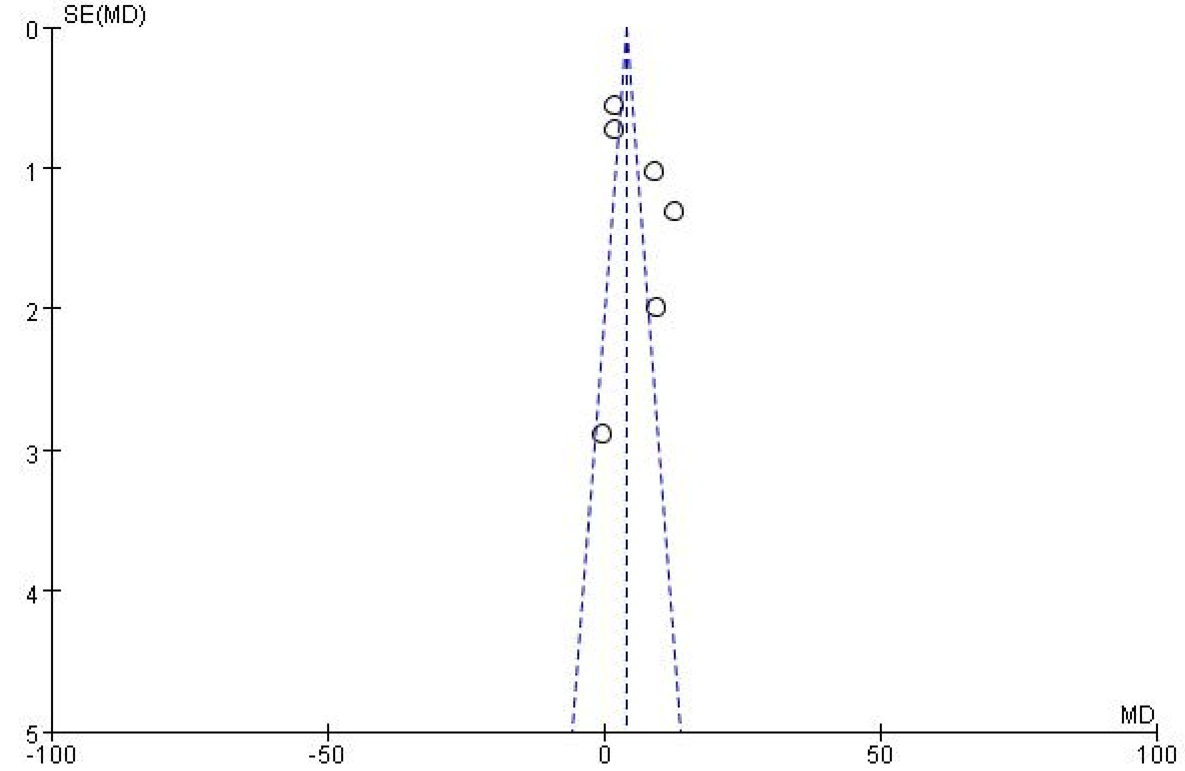

**Figure S4. CD4+ T cells funnel plot**

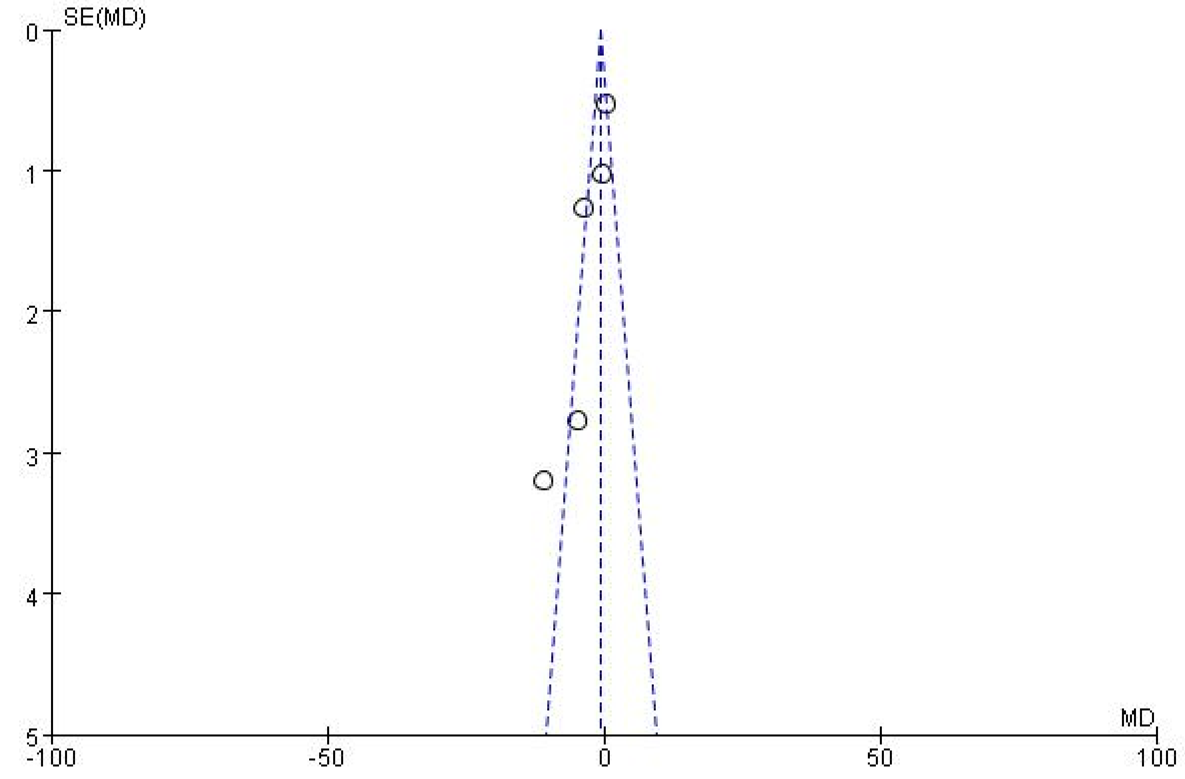


**Figure S5. CD8+ T cells funnel plot**

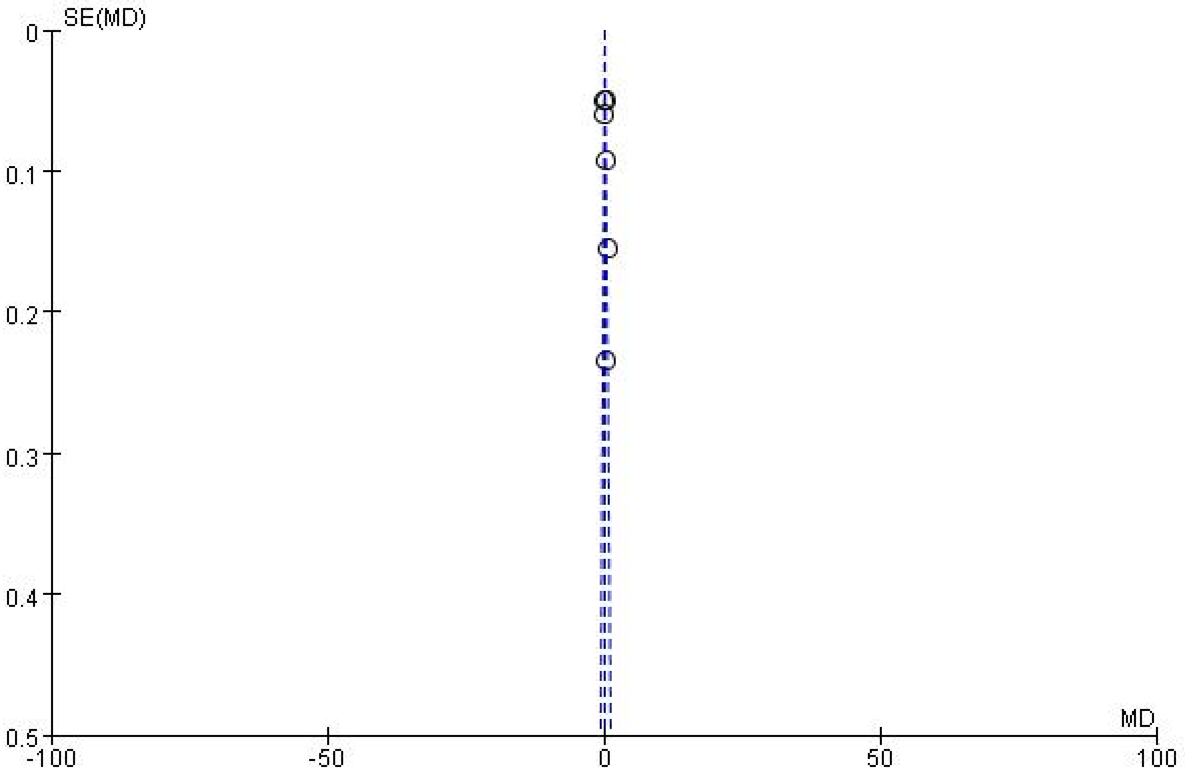

**Figure S6. CD4+CD8+ T cells funnel plot**

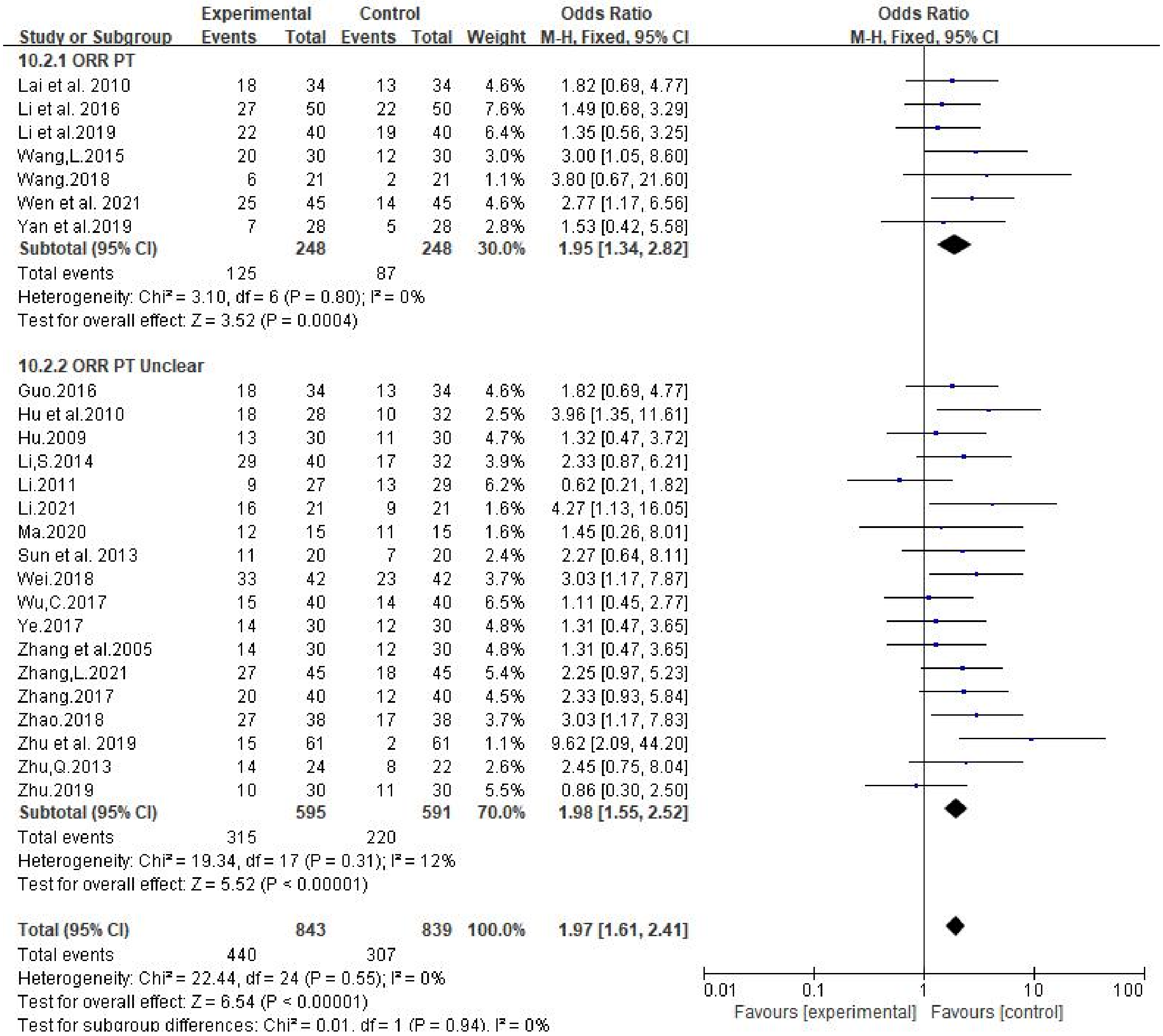

| Study or Subgroup | Experimental Events | Experimental Total | Control Events | Control Total | Weight | Odds Ratio M-H, Fixed, 95% CI |
|---|---|---|---|---|---|---|
| **10.2.1 ORR PT** | | | | | | |
| Lai et al. 2010 | 18 | 34 | 13 | 34 | 4.6% | 1.82 [0.69, 4.77] |
| Li et al. 2016 | 27 | 50 | 22 | 50 | 7.6% | 1.49 [0.68, 3.29] |
| Li et al.2019 | 22 | 40 | 19 | 40 | 6.4% | 1.35 [0.56, 3.25] |
| Wang,L.2015 | 20 | 30 | 12 | 30 | 3.0% | 3.00 [1.05, 8.60] |
| Wang.2018 | 6 | 21 | 2 | 21 | 1.1% | 3.80 [0.67, 21.60] |
| Wen et al. 2021 | 25 | 45 | 14 | 45 | 4.6% | 2.77 [1.17, 6.56] |
| Yan et al.2019 | 7 | 28 | 5 | 28 | 2.8% | 1.53 [0.42, 5.58] |
| **Subtotal (95% CI)** | | **248** | | **248** | **30.0%** | **1.95 [1.34, 2.82]** |
| Total events | 125 | | 87 | | | |
| Heterogeneity: Chi² = 3.10, df = 6 (P = 0.80); I² = 0% | | | | | | |
| Test for overall effect: Z = 3.52 (P = 0.0004) | | | | | | |
| **10.2.2 ORR PT Unclear** | | | | | | |
| Guo.2016 | 18 | 34 | 13 | 34 | 4.6% | 1.82 [0.69, 4.77] |
| Hu et al.2010 | 18 | 28 | 10 | 32 | 2.5% | 3.96 [1.35, 11.61] |
| Hu.2009 | 13 | 30 | 11 | 30 | 4.7% | 1.32 [0.47, 3.72] |
| Li,S.2014 | 29 | 40 | 17 | 32 | 3.9% | 2.33 [0.87, 6.21] |
| Li.2011 | 9 | 27 | 13 | 29 | 6.2% | 0.62 [0.21, 1.82] |
| Li.2021 | 16 | 21 | 9 | 21 | 1.6% | 4.27 [1.13, 16.05] |
| Ma.2020 | 12 | 15 | 11 | 15 | 1.6% | 1.45 [0.26, 8.01] |
| Sun et al. 2013 | 11 | 20 | 7 | 20 | 2.4% | 2.27 [0.64, 8.11] |
| Wei.2018 | 33 | 42 | 23 | 42 | 3.7% | 3.03 [1.17, 7.87] |
| Wu,C.2017 | 15 | 40 | 14 | 40 | 6.5% | 1.11 [0.45, 2.77] |
| Ye.2017 | 14 | 30 | 12 | 30 | 4.8% | 1.31 [0.47, 3.65] |
| Zhang et al.2005 | 14 | 30 | 12 | 30 | 4.8% | 1.31 [0.47, 3.65] |
| Zhang,L.2021 | 27 | 45 | 18 | 45 | 5.4% | 2.25 [0.97, 5.23] |
| Zhang.2017 | 20 | 40 | 12 | 40 | 4.5% | 2.33 [0.93, 5.84] |
| Zhao.2018 | 27 | 38 | 17 | 38 | 3.7% | 3.03 [1.17, 7.83] |
| Zhu et al. 2019 | 15 | 61 | 2 | 61 | 1.1% | 9.62 [2.09, 44.20] |
| Zhu,Q.2013 | 14 | 24 | 8 | 22 | 2.6% | 2.45 [0.75, 8.04] |
| Zhu.2019 | 10 | 30 | 11 | 30 | 5.5% | 0.86 [0.30, 2.50] |
| **Subtotal (95% CI)** | | **595** | | **591** | **70.0%** | **1.98 [1.55, 2.52]** |
| Total events | 315 | | 220 | | | |
| Heterogeneity: Chi² = 19.34, df = 17 (P = 0.31); I² = 12% | | | | | | |
| Test for overall effect: Z = 5.52 (P < 0.00001) | | | | | | |
| **Total (95% CI)** | | **843** | | **839** | **100.0%** | **1.97 [1.61, 2.41]** |
| Total events | 440 | | 307 | | | |
| Heterogeneity: Chi² = 22.44, df = 24 (P = 0.55); I² = 0% | | | | | | |
| Test for overall effect: Z = 6.54 (P < 0.00001) | | | | | | |
| Test for subgroup differences: Chi² = 0.01, df = 1 (P = 0.94), I² = 0% | | | | | | |



**Figure S7. Subgroup analysis of ORR therapy procedure**

| Study or Subgroup | Experimental Events | Experimental Total | Control Events | Control Total | Weight | Odds Ratio M-H, Fixed, 95% CI |
|---|---|---|---|---|---|---|
| **10.15.1 Objective response rate( S-1-based chemotherapy regimen** | | | | | | |
| Li et al. 2016 | 27 | 50 | 22 | 50 | 8.4% | 1.49 [0.68, 3.29] |
| Ma.2020 | 12 | 15 | 11 | 15 | 1.8% | 1.45 [0.26, 8.01] |
| Sun et al. 2013 | 11 | 20 | 7 | 20 | 2.6% | 2.27 [0.64, 8.11] |
| Wang.2018 | 6 | 21 | 2 | 21 | 1.2% | 3.80 [0.67, 21.60] |
| Wu,C.2017 | 15 | 40 | 14 | 40 | 7.3% | 1.11 [0.45, 2.77] |
| Zhang.2017 | 20 | 40 | 12 | 40 | 5.0% | 2.33 [0.93, 5.84] |
| Zhu.2019 | 10 | 30 | 11 | 30 | 6.1% | 0.86 [0.30, 2.50] |
| **Subtotal (95% CI)** | | **216** | | **216** | **32.4%** | **1.56 [1.05, 2.33]** |
| Total events | 101 | | 79 | | | |
| Heterogeneity: Chi² = 3.81, df = 6 (P = 0.70); I² = 0% | | | | | | |
| Test for overall effect: Z = 2.21 (P = 0.03) | | | | | | |
| **10.15.2 Objective response rate( 5 Fu-based chemotherapy regimen)** | | | | | | |
| Guo.2016 | 18 | 34 | 13 | 34 | 5.1% | 1.82 [0.69, 4.77] |
| Hu et al.2010 | 18 | 28 | 10 | 32 | 2.8% | 3.96 [1.35, 11.61] |
| Hu.2009 | 13 | 30 | 11 | 30 | 5.2% | 1.32 [0.47, 3.72] |
| Lai et al. 2010 | 13 | 25 | 13 | 30 | 4.7% | 1.42 [0.49, 4.12] |
| Li et al.2019 | 22 | 40 | 19 | 40 | 7.1% | 1.35 [0.56, 3.25] |
| Li.2011 | 9 | 27 | 13 | 29 | 6.9% | 0.62 [0.21, 1.82] |
| Wei.2018 | 33 | 42 | 23 | 42 | 4.1% | 3.03 [1.17, 7.87] |
| Wen et al. 2021 | 25 | 45 | 14 | 45 | 5.2% | 2.77 [1.17, 6.56] |
| Yan et al.2019 | 7 | 28 | 5 | 28 | 3.1% | 1.53 [0.42, 5.58] |
| Zhang et al.2005 | 14 | 30 | 12 | 30 | 5.3% | 1.31 [0.47, 3.65] |
| Zhao.2018 | 27 | 38 | 17 | 38 | 4.1% | 3.03 [1.17, 7.83] |
| Zhu,Q.2013 | 14 | 24 | 8 | 22 | 2.9% | 2.45 [0.75, 8.04] |
| **Subtotal (95% CI)** | | **391** | | **400** | **56.5%** | **1.87 [1.40, 2.49]** |
| Total events | 213 | | 158 | | | |
| Heterogeneity: Chi² = 10.65, df = 11 (P = 0.47); I² = 0% | | | | | | |
| Test for overall effect: Z = 4.26 (P < 0.0001) | | | | | | |
| **10.15.3 Objective response rate( CAP-based chemotherapy regimen)** | | | | | | |
| Li.2021 | 16 | 21 | 9 | 21 | 1.8% | 4.27 [1.13, 16.05] |
| Wang,L.2015 | 20 | 30 | 12 | 30 | 3.3% | 3.00 [1.05, 8.60] |
| Zhang,L.2021 | 27 | 45 | 18 | 45 | 6.0% | 2.25 [0.97, 5.23] |
| **Subtotal (95% CI)** | | **96** | | **96** | **11.1%** | **2.80 [1.56, 5.04]** |
| Total events | 63 | | 39 | | | |
| Heterogeneity: Chi² = 0.66, df = 2 (P = 0.72); I² = 0% | | | | | | |
| Test for overall effect: Z = 3.43 (P = 0.0006) | | | | | | |
| **Total (95% CI)** | | **703** | | **712** | **100.0%** | **1.87 [1.51, 2.33]** |
| Total events | 377 | | 276 | | | |
| Heterogeneity: Chi² = 17.71, df = 21 (P = 0.67); I² = 0% | | | | | | |
| Test for overall effect: Z = 5.69 (P < 0.00001) | | | | | | |
| Test for subgroup differences: Chi² = 2.58, df = 2 (P = 0.27), I² = 22.6% | | | | | | |

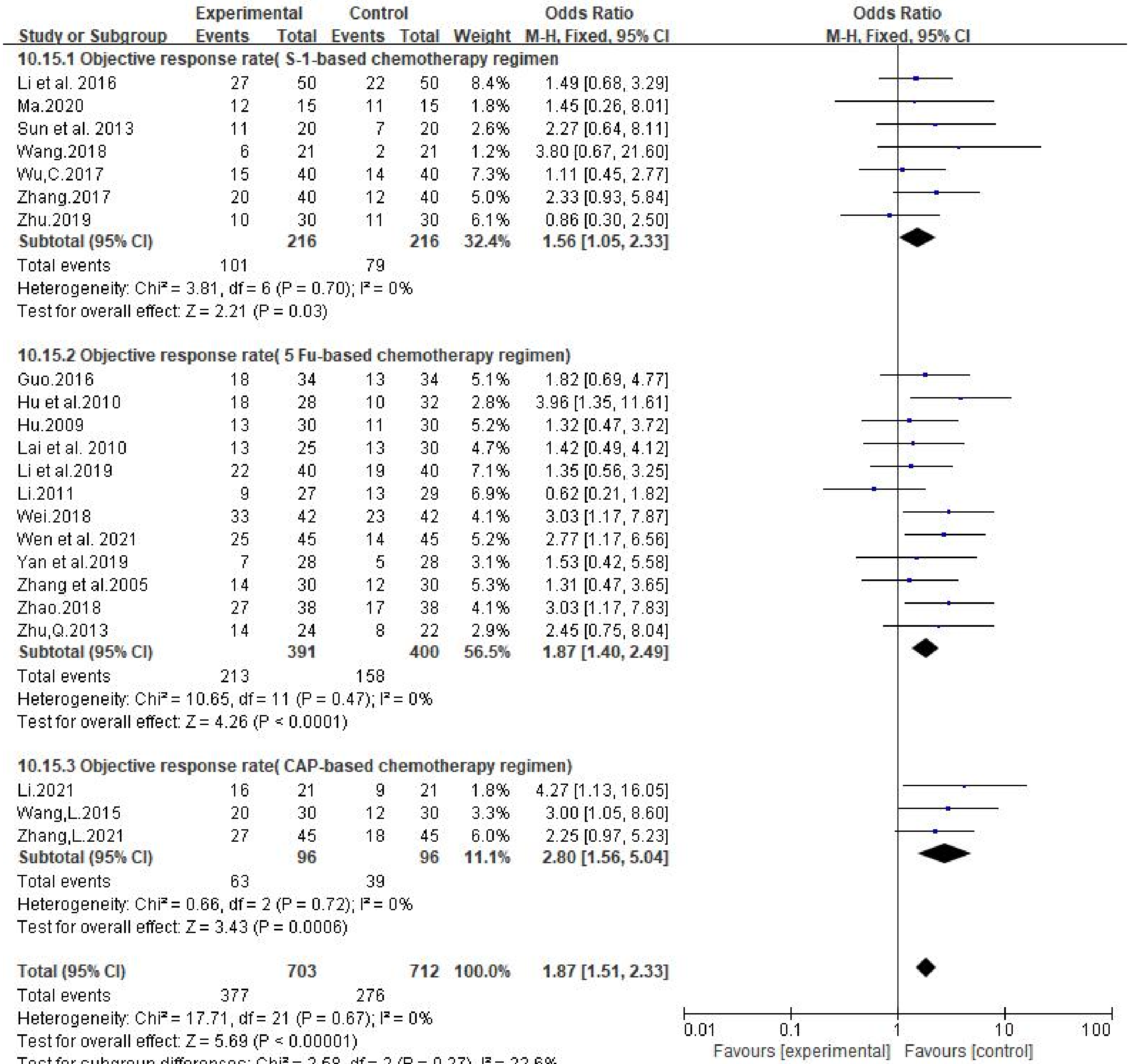


**Figure S8. ORR Subgroup Analysis of Chemotherapy Regimens Using Fluoropyrimidine**

| Study or Subgroup | Experimental Events | Experimental Total | Control Events | Control Total | Weight | Odds Ratio M-H, Fixed, 95% CI |
|---|---|---|---|---|---|---|
| **10.4.1 Objective response rate( OXA-based chemotherapy regimen)** | | | | | | |
| Guo.2016 | 18 | 34 | 13 | 34 | 6.0% | 1.82 [0.69, 4.77] |
| Hu et al.2010 | 18 | 28 | 10 | 32 | 3.3% | 3.96 [1.35, 11.61] |
| Hu.2009 | 13 | 30 | 11 | 30 | 6.1% | 1.32 [0.47, 3.72] |
| Li,S.2014 | 29 | 40 | 17 | 32 | 5.1% | 2.33 [0.87, 6.21] |
| Li.2021 | 16 | 21 | 9 | 21 | 2.1% | 4.27 [1.13, 16.05] |
| Ma.2020 | 12 | 15 | 11 | 15 | 2.2% | 1.45 [0.26, 8.01] |
| Wang.2018 | 6 | 21 | 2 | 21 | 1.4% | 3.80 [0.67, 21.60] |
| Wei.2018 | 33 | 42 | 23 | 42 | 4.8% | 3.03 [1.17, 7.87] |
| Wen et al. 2021 | 25 | 45 | 14 | 45 | 6.1% | 2.77 [1.17, 6.56] |
| Wu,C.2017 | 15 | 40 | 14 | 40 | 8.6% | 1.11 [0.45, 2.77] |
| Ye.2017 | 14 | 30 | 12 | 30 | 6.3% | 1.31 [0.47, 3.65] |
| Zhang,L.2021 | 27 | 45 | 18 | 45 | 7.1% | 2.25 [0.97, 5.23] |
| Zhu et al. 2019 | 15 | 61 | 2 | 61 | 1.5% | 9.62 [2.09, 44.20] |
| Zhu.2019 | 10 | 30 | 11 | 30 | 7.2% | 0.86 [0.30, 2.50] |
| **Subtotal (95% CI)** | | **482** | | **478** | **67.6%** | **2.17 [1.65, 2.86]** |
| Total events | 251 | | 167 | | | |
| Heterogeneity: Chi² = 14.16, df = 13 (P = 0.36); I² = 8% | | | | | | |
| Test for overall effect: Z = 5.49 (P < 0.00001) | | | | | | |
| **10.4.2 Objective response rate( DDP-based chemotherapy regimen)** | | | | | | |
| Li et al.2019 | 22 | 40 | 19 | 40 | 8.4% | 1.35 [0.56, 3.25] |
| Li.2011 | 9 | 27 | 13 | 29 | 8.2% | 0.62 [0.21, 1.82] |
| Wang,L.2015 | 20 | 30 | 12 | 30 | 3.9% | 3.00 [1.05, 8.60] |
| Yan et al.2019 | 7 | 28 | 5 | 28 | 3.7% | 1.53 [0.42, 5.58] |
| Zhao.2018 | 27 | 38 | 17 | 38 | 4.8% | 3.03 [1.17, 7.83] |
| Zhu,Q.2013 | 14 | 24 | 8 | 22 | 3.4% | 2.45 [0.75, 8.04] |
| **Subtotal (95% CI)** | | **187** | | **187** | **32.4%** | **1.75 [1.15, 2.66]** |
| Total events | 99 | | 74 | | | |
| Heterogeneity: Chi² = 6.54, df = 5 (P = 0.26); I² = 24% | | | | | | |
| Test for overall effect: Z = 2.62 (P = 0.009) | | | | | | |
| **Total (95% CI)** | | **669** | | **665** | **100.0%** | **2.03 [1.62, 2.56]** |
| Total events | 350 | | 241 | | | |
| Heterogeneity: Chi² = 21.17, df = 19 (P = 0.33); I² = 10% | | | | | | |
| Test for overall effect: Z = 6.04 (P < 0.00001) | | | | | | |
| Test for subgroup differences: Chi² = 0.70, df = 1 (P = 0.40), I² = 0% | | | | | | |

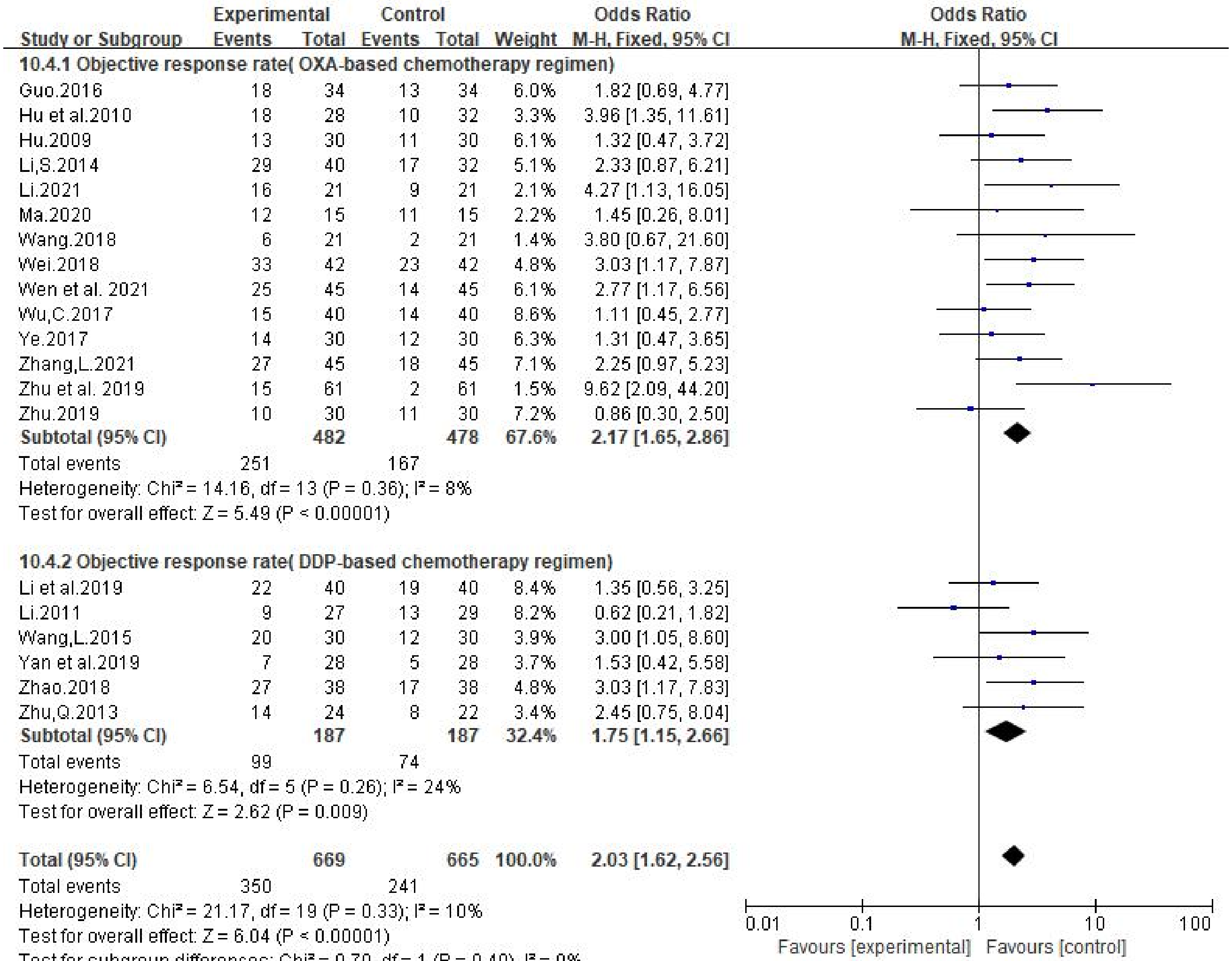


**Figure S9. ORR Subgroup Analysis of Chemotherapy Regimens Using platinum**

| Study or Subgroup | Experimental Events | Experimental Total | Control Events | Control Total | Weight | Odds Ratio M-H, Fixed, 95% CI |
|---|---|---|---|---|---|---|
| **10.6.1 Objective response rate( <8 weeks)** | | | | | | |
| Li et al.2019 | 22 | 40 | 19 | 40 | 6.7% | 1.35 [0.56, 3.25] |
| Li,S.2014 | 29 | 40 | 17 | 32 | 4.0% | 2.33 [0.87, 6.21] |
| Wei.2018 | 33 | 42 | 23 | 42 | 3.8% | 3.03 [1.17, 7.87] |
| Wen et al. 2021 | 25 | 45 | 14 | 45 | 4.8% | 2.77 [1.17, 6.56] |
| Yan et al.2019 | 7 | 28 | 5 | 28 | 2.9% | 1.53 [0.42, 5.58] |
| Zhang et al.2005 | 14 | 30 | 12 | 30 | 5.0% | 1.31 [0.47, 3.65] |
| Zhu,Q.2013 | 14 | 24 | 8 | 22 | 2.7% | 2.45 [0.75, 8.04] |
| **Subtotal (95% CI)** | | **249** | | **239** | **30.0%** | **2.04 [1.40, 2.96]** |
| Total events | 144 | | 98 | | | |
| Heterogeneity: Chi² = 3.04, df = 6 (P = 0.80); I² = 0% | | | | | | |
| Test for overall effect: Z = 3.72 (P = 0.0002) | | | | | | |
| **10.6.2 Objective response rate( ⩾8 weeks)** | | | | | | |
| Guo.2016 | 18 | 34 | 13 | 34 | 4.8% | 1.82 [0.69, 4.77] |
| Hu et al.2010 | 18 | 28 | 10 | 32 | 2.6% | 3.96 [1.35, 11.61] |
| Hu.2009 | 13 | 30 | 11 | 30 | 4.9% | 1.32 [0.47, 3.72] |
| Li,S.2014 | 29 | 40 | 17 | 32 | 4.0% | 2.33 [0.87, 6.21] |
| Li.2011 | 9 | 27 | 13 | 29 | 6.5% | 0.62 [0.21, 1.82] |
| Li.2021 | 16 | 21 | 9 | 21 | 1.7% | 4.27 [1.13, 16.05] |
| Ma.2020 | 12 | 15 | 11 | 15 | 1.7% | 1.45 [0.26, 8.01] |
| Sun et al. 2013 | 11 | 20 | 7 | 20 | 2.5% | 2.27 [0.64, 8.11] |
| Wei.2018 | 6 | 21 | 2 | 21 | 1.1% | 3.80 [0.67, 21.60] |
| Wen et al. 2021 | 25 | 45 | 14 | 45 | 4.8% | 2.77 [1.17, 6.56] |
| Wu,C.2017 | 15 | 40 | 14 | 40 | 6.8% | 1.11 [0.45, 2.77] |
| Ye.2017 | 14 | 30 | 12 | 30 | 5.0% | 1.31 [0.47, 3.65] |
| Zhang,L.2021 | 27 | 45 | 18 | 45 | 5.6% | 2.25 [0.97, 5.23] |
| Zhang.2017 | 20 | 40 | 12 | 40 | 4.7% | 2.33 [0.93, 5.84] |
| Zhao.2018 | 27 | 38 | 17 | 38 | 3.8% | 3.03 [1.17, 7.83] |
| Zhu et al. 2019 | 15 | 61 | 2 | 61 | 1.2% | 9.62 [2.09, 44.20] |
| Zhu,Q.2013 | 14 | 24 | 8 | 22 | 2.7% | 2.45 [0.75, 8.04] |
| Zhu.2019 | 10 | 30 | 11 | 30 | 5.7% | 0.86 [0.30, 2.50] |
| **Subtotal (95% CI)** | | **589** | | **585** | **70.0%** | **2.05 [1.61, 2.62]** |
| Total events | 299 | | 201 | | | |
| Heterogeneity: Chi² = 19.10, df = 17 (P = 0.32); I² = 11% | | | | | | |
| Test for overall effect: Z = 5.74 (P < 0.00001) | | | | | | |
| **Total (95% CI)** | | **838** | | **824** | **100.0%** | **2.05 [1.67, 2.51]** |
| Total events | 443 | | 299 | | | |
| Heterogeneity: Chi² = 22.13, df = 24 (P = 0.57); I² = 0% | | | | | | |
| Test for overall effect: Z = 6.85 (P < 0.00001) | | | | | | |
| Test for subgroup differences: Chi² = 0.00, df = 1 (P = 0.97), I² = 0% | | | | | | |

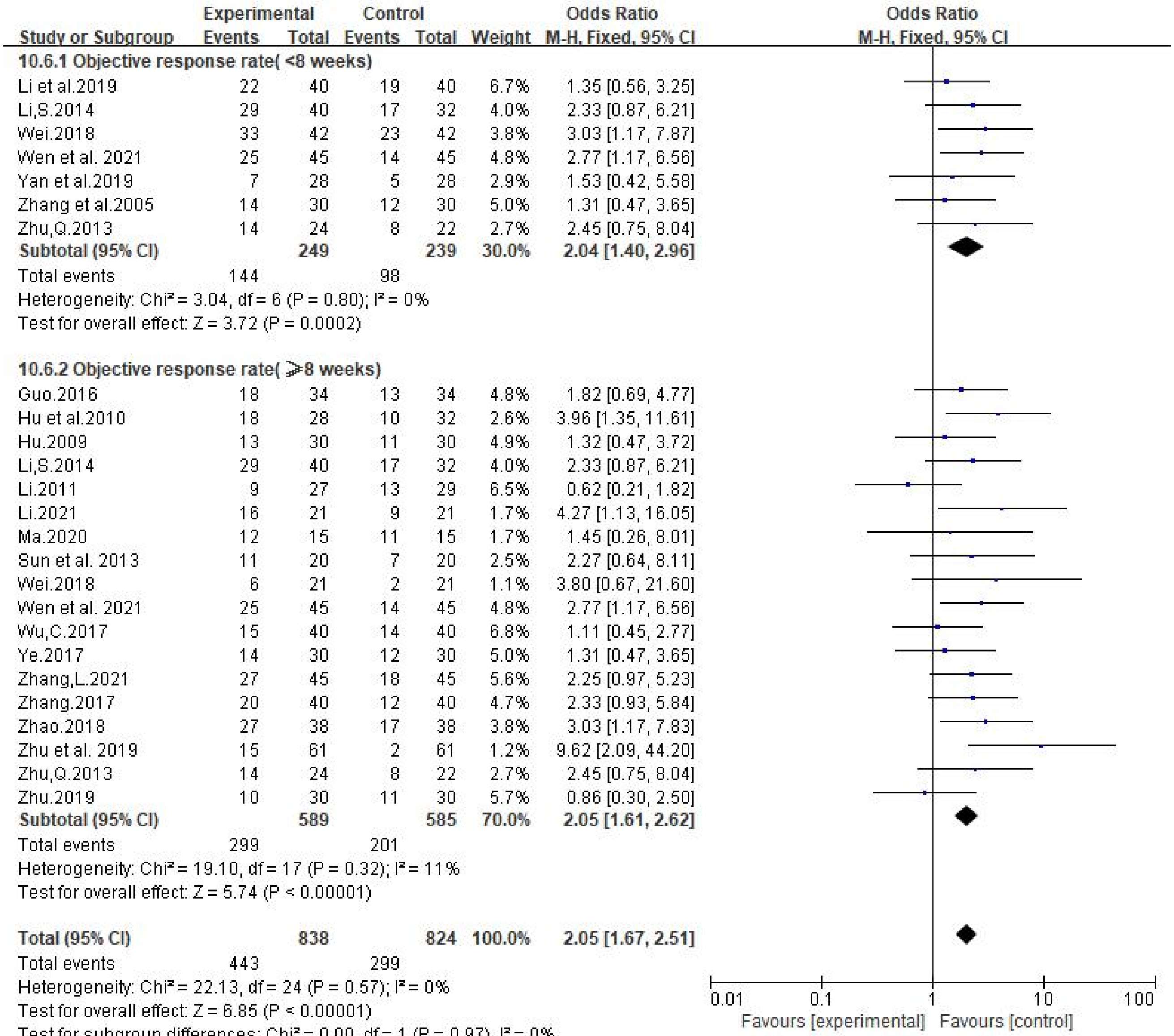


**Figure S10. Subgroup analysis of ORR via treatment duration**

| Study or Subgroup | Experimental Events | Experimental Total | Control Events | Control Total | Weight | Odds Ratio M-H, Fixed, 95% CI |
|---|---|---|---|---|---|---|
| **10.8.1 Disease Control Rate PT** | | | | | | |
| Lai et al. 2010 | 18 | 34 | 13 | 34 | 4.9% | 1.82 [0.69, 4.77] |
| Li et al. 2016 | 27 | 50 | 22 | 50 | 8.1% | 1.49 [0.68, 3.29] |
| Li et al.2019 | 22 | 40 | 19 | 40 | 6.8% | 1.35 [0.56, 3.25] |
| Wang,L.2015 | 20 | 30 | 12 | 30 | 3.2% | 3.00 [1.05, 8.60] |
| Wang.2018 | 6 | 21 | 2 | 21 | 1.1% | 3.80 [0.67, 21.60] |
| Wen et al. 2021 | 41 | 45 | 33 | 45 | 2.3% | 3.73 [1.10, 12.64] |
| Yan et al.2019 | 7 | 28 | 5 | 28 | 3.0% | 1.53 [0.42, 5.58] |
| **Subtotal (95% CI)** | | **248** | | **248** | **29.4%** | **1.95 [1.32, 2.87]** |
| Total events | 141 | | 106 | | | |
| Heterogeneity: Chi² = 3.55, df = 6 (P = 0.74); I² = 0% | | | | | | |
| Test for overall effect: Z = 3.37 (P = 0.0008) | | | | | | |
| **10.8.2 Disease Control Rate PT Unclear** | | | | | | |
| Guo.2016 | 18 | 34 | 13 | 34 | 4.9% | 1.82 [0.69, 4.77] |
| Hu et al.2010 | 18 | 28 | 10 | 32 | 2.7% | 3.96 [1.35, 11.61] |
| Hu.2009 | 13 | 30 | 11 | 30 | 5.0% | 1.32 [0.47, 3.72] |
| Li,S.2014 | 29 | 40 | 17 | 32 | 4.1% | 2.33 [0.87, 6.21] |
| Li.2011 | 9 | 27 | 13 | 29 | 6.7% | 0.62 [0.21, 1.82] |
| Li.2021 | 16 | 21 | 9 | 21 | 1.7% | 4.27 [1.13, 16.05] |
| Ma.2020 | 12 | 15 | 11 | 15 | 1.8% | 1.45 [0.26, 8.01] |
| Sun et al. 2013 | 11 | 20 | 7 | 20 | 2.5% | 2.27 [0.64, 8.11] |
| Wu,C.2017 | 15 | 40 | 14 | 40 | 7.0% | 1.11 [0.45, 2.77] |
| Ye.2017 | 14 | 30 | 12 | 30 | 5.1% | 1.31 [0.47, 3.65] |
| Zhang et al.2005 | 14 | 30 | 12 | 30 | 5.1% | 1.31 [0.47, 3.65] |
| Zhang,L.2021 | 27 | 45 | 18 | 45 | 5.7% | 2.25 [0.97, 5.23] |
| Zhang.2017 | 20 | 40 | 12 | 40 | 4.8% | 2.33 [0.93, 5.84] |
| Zhao.2018 | 27 | 38 | 17 | 38 | 3.9% | 3.03 [1.17, 7.83] |
| Zhu et al. 2019 | 15 | 61 | 2 | 61 | 1.2% | 9.62 [2.09, 44.20] |
| Zhu,Q.2013 | 14 | 24 | 8 | 22 | 2.8% | 2.45 [0.75, 8.04] |
| Zhu.2019 | 10 | 30 | 11 | 30 | 5.8% | 0.86 [0.30, 2.50] |
| **Subtotal (95% CI)** | | **553** | | **549** | **70.6%** | **1.92 [1.49, 2.47]** |
| Total events | 282 | | 197 | | | |
| Heterogeneity: Chi² = 18.45, df = 16 (P = 0.30); I² = 13% | | | | | | |
| Test for overall effect: Z = 5.10 (P < 0.00001) | | | | | | |
| **Total (95% CI)** | | **801** | | **797** | **100.0%** | **1.93 [1.56, 2.38]** |
| Total events | 423 | | 303 | | | |
| Heterogeneity: Chi² = 22.01, df = 23 (P = 0.52); I² = 0% | | | | | | |
| Test for overall effect: Z = 6.11 (P < 0.00001) | | | | | | |
| Test for subgroup differences: Chi² = 0.00, df = 1 (P = 0.95), I² = 0% | | | | | | |

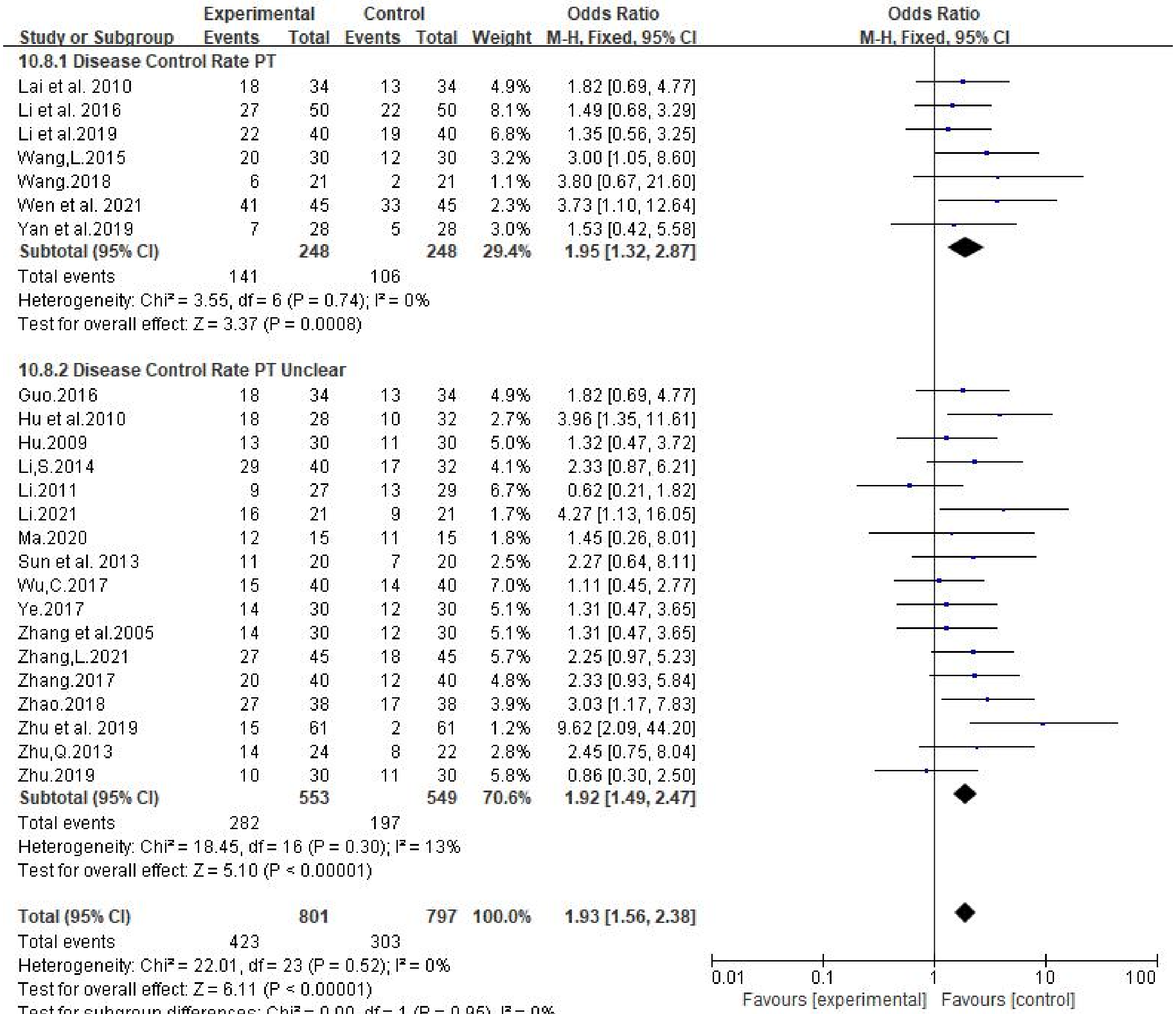


**Figure S11. Subgroup analysis of DCR therapy procedure**

| Study or Subgroup | Experimental Events | Experimental Total | Control Events | Control Total | Weight | Odds Ratio M-H, Fixed, 95% CI |
|---|---|---|---|---|---|---|
| **10.13.1 Disease Control Rate( S-1-based chemotherapy regimen)** | | | | | | |
| Li et al. 2016 | 27 | 50 | 22 | 50 | 8.8% | 1.49 [0.68, 3.29] |
| Ma.2020 | 12 | 15 | 11 | 15 | 1.9% | 1.45 [0.26, 8.01] |
| Sun et al. 2013 | 11 | 20 | 7 | 20 | 2.7% | 2.27 [0.64, 8.11] |
| Wang.2018 | 6 | 21 | 2 | 21 | 1.2% | 3.80 [0.67, 21.60] |
| Wu,C.2017 | 15 | 40 | 14 | 40 | 7.6% | 1.11 [0.45, 2.77] |
| Zhang.2017 | 20 | 40 | 12 | 40 | 5.2% | 2.33 [0.93, 5.84] |
| Zhu.2019 | 10 | 30 | 11 | 30 | 6.4% | 0.86 [0.30, 2.50] |
| **Subtotal (95% CI)** | | **216** | | **216** | **33.8%** | **1.56 [1.05, 2.33]** |
| Total events | 101 | | 79 | | | |
| Heterogeneity: Chi² = 3.81, df = 6 (P = 0.70); I² = 0% | | | | | | |
| Test for overall effect: Z = 2.21 (P = 0.03) | | | | | | |
| **10.13.2 Disease Control Rate( 5 Fu-based chemotherapy regimen)** | | | | | | |
| Guo.2016 | 18 | 34 | 13 | 34 | 5.3% | 1.82 [0.69, 4.77] |
| Hu et al.2010 | 18 | 28 | 10 | 32 | 2.9% | 3.96 [1.35, 11.61] |
| Hu.2009 | 13 | 30 | 11 | 30 | 5.4% | 1.32 [0.47, 3.72] |
| Lai et al. 2010 | 13 | 25 | 13 | 30 | 4.9% | 1.42 [0.49, 4.12] |
| Li et al.2019 | 22 | 40 | 19 | 40 | 7.4% | 1.35 [0.56, 3.25] |
| Li.2011 | 9 | 27 | 13 | 29 | 7.2% | 0.62 [0.21, 1.82] |
| Wen et al. 2021 | 25 | 45 | 14 | 45 | 5.4% | 2.77 [1.17, 6.56] |
| Yan et al.2019 | 7 | 28 | 5 | 28 | 3.3% | 1.53 [0.42, 5.58] |
| Zhang et al.2005 | 14 | 30 | 12 | 30 | 5.5% | 1.31 [0.47, 3.65] |
| Zhao.2018 | 27 | 38 | 17 | 38 | 4.3% | 3.03 [1.17, 7.83] |
| Zhu,Q.2013 | 14 | 24 | 8 | 22 | 3.0% | 2.45 [0.75, 8.04] |
| **Subtotal (95% CI)** | | **349** | | **358** | **54.6%** | **1.78 [1.31, 2.41]** |
| Total events | 180 | | 135 | | | |
| Heterogeneity: Chi² = 9.57, df = 10 (P = 0.48); I² = 0% | | | | | | |
| Test for overall effect: Z = 3.73 (P = 0.0002) | | | | | | |
| **10.13.3 Disease Control Rate( CAP-based chemotherapy regimen)** | | | | | | |
| Li.2021 | 16 | 21 | 9 | 21 | 1.9% | 4.27 [1.13, 16.05] |
| Wang,L.2015 | 20 | 30 | 12 | 30 | 3.5% | 3.00 [1.05, 8.60] |
| Zhang,L.2021 | 27 | 45 | 18 | 45 | 6.2% | 2.25 [0.97, 5.23] |
| **Subtotal (95% CI)** | | **96** | | **96** | **11.6%** | **2.80 [1.56, 5.04]** |
| Total events | 63 | | 39 | | | |
| Heterogeneity: Chi² = 0.66, df = 2 (P = 0.72); I² = 0% | | | | | | |
| Test for overall effect: Z = 3.43 (P = 0.0006) | | | | | | |
| **Total (95% CI)** | | **661** | | **670** | **100.0%** | **1.82 [1.46, 2.28]** |
| Total events | 344 | | 253 | | | |
| Heterogeneity: Chi² = 16.68, df = 20 (P = 0.67); I² = 0% | | | | | | |
| Test for overall effect: Z = 5.30 (P < 0.00001) | | | | | | |
| Test for subgroup differences: Chi² = 2.64, df = 2 (P = 0.27), I² = 24.3% | | | | | | |

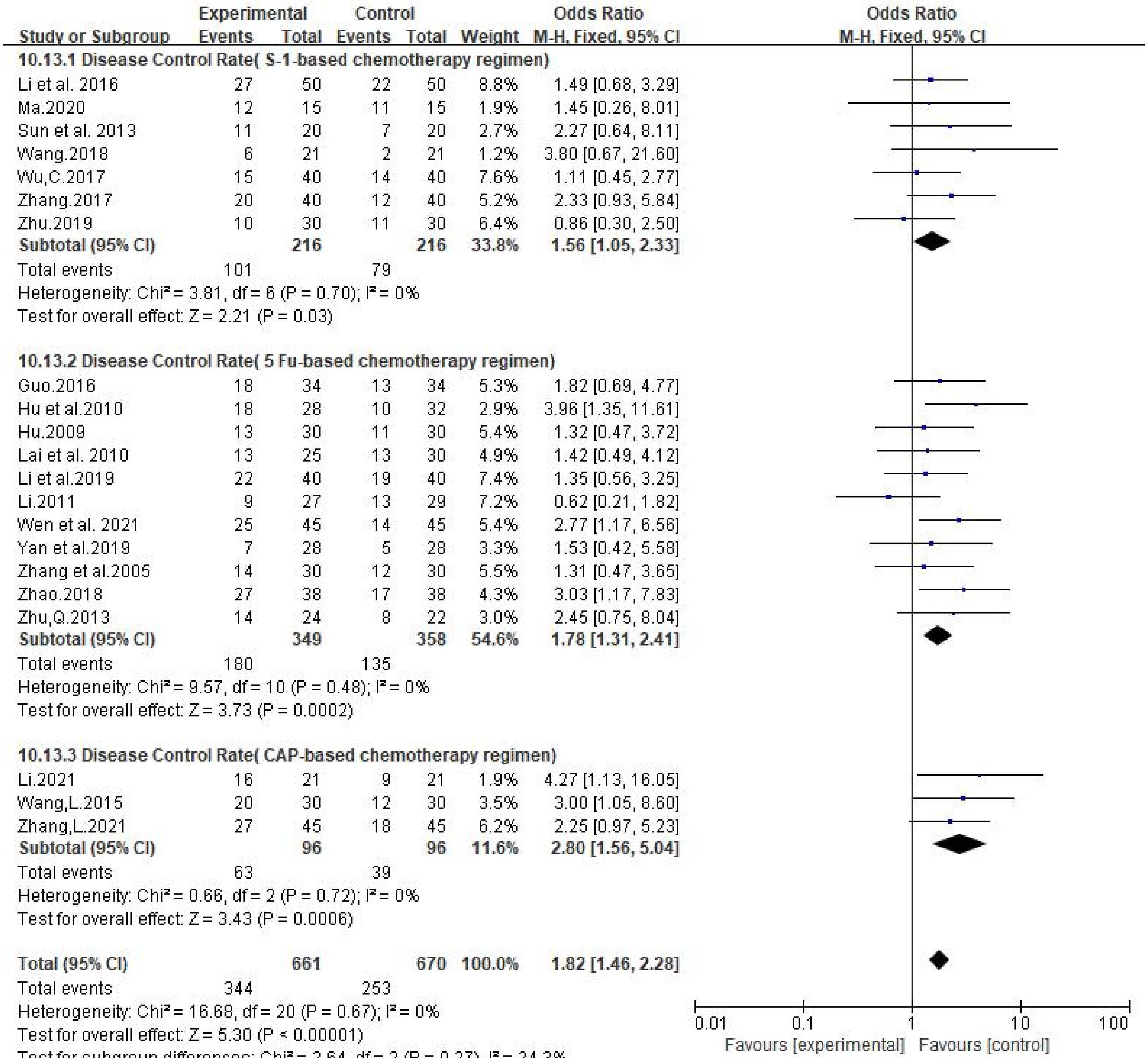


**Figure S12. DCR Subgroup Analysis of Chemotherapy Regimens Using Fluoropyrimidine**

| Study or Subgroup | Experimental Events | Experimental Total | Control Events | Control Total | Weight | Odds Ratio M-H, Fixed, 95% CI |
|---|---|---|---|---|---|---|
| **10.18.1 Disease Control Rate( OXA-based chemotherapy regimen)** | | | | | | |
| Guo.2016 | 18 | 34 | 13 | 34 | 6.3% | 1.82 [0.69, 4.77] |
| Hu et al.2010 | 18 | 28 | 10 | 32 | 3.4% | 3.96 [1.35, 11.61] |
| Hu.2009 | 13 | 30 | 11 | 30 | 6.4% | 1.32 [0.47, 3.72] |
| Li,S.2014 | 29 | 40 | 17 | 32 | 5.3% | 2.33 [0.87, 6.21] |
| Li.2021 | 16 | 21 | 9 | 21 | 2.2% | 4.27 [1.13, 16.05] |
| Ma.2020 | 12 | 15 | 11 | 15 | 2.3% | 1.45 [0.26, 8.01] |
| Wang.2018 | 6 | 21 | 2 | 21 | 1.5% | 3.80 [0.67, 21.60] |
| Wen et al. 2021 | 25 | 45 | 14 | 45 | 6.4% | 2.77 [1.17, 6.56] |
| Wu,C.2017 | 15 | 40 | 14 | 40 | 9.0% | 1.11 [0.45, 2.77] |
| Ye.2017 | 14 | 30 | 12 | 30 | 6.6% | 1.31 [0.47, 3.65] |
| Zhang,L.2021 | 27 | 45 | 18 | 45 | 7.4% | 2.25 [0.97, 5.23] |
| Zhu et al. 2019 | 15 | 61 | 2 | 61 | 1.6% | 9.62 [2.09, 44.20] |
| Zhu.2019 | 10 | 30 | 11 | 30 | 7.6% | 0.86 [0.30, 2.50] |
| **Subtotal (95% CI)** | | **440** | | **436** | **66.0%** | **2.10 [1.58, 2.81]** |
| Total events | 218 | | 144 | | | |
| Heterogeneity: Chi² = 13.56, df = 12 (P = 0.33); I² = 12% | | | | | | |
| Test for overall effect: Z = 5.05 (P < 0.00001) | | | | | | |
| **10.18.2 Disease Control Rate( DDP-based chemotherapy regimen)** | | | | | | |
| Li et al.2019 | 22 | 40 | 19 | 40 | 8.8% | 1.35 [0.56, 3.25] |
| Li.2011 | 9 | 27 | 13 | 29 | 8.6% | 0.62 [0.21, 1.82] |
| Wang,L.2015 | 20 | 30 | 12 | 30 | 4.1% | 3.00 [1.05, 8.60] |
| Yan et al.2019 | 7 | 28 | 5 | 28 | 3.9% | 1.53 [0.42, 5.58] |
| Zhao.2018 | 27 | 38 | 17 | 38 | 5.1% | 3.03 [1.17, 7.83] |
| Zhu,Q.2013 | 14 | 24 | 8 | 22 | 3.6% | 2.45 [0.75, 8.04] |
| **Subtotal (95% CI)** | | **187** | | **187** | **34.0%** | **1.75 [1.15, 2.66]** |
| Total events | 99 | | 74 | | | |
| Heterogeneity: Chi² = 6.54, df = 5 (P = 0.26); I² = 24% | | | | | | |
| Test for overall effect: Z = 2.62 (P = 0.009) | | | | | | |
| **Total (95% CI)** | | **627** | | **623** | **100.0%** | **1.98 [1.56, 2.52]** |
| Total events | 317 | | 218 | | | |
| Heterogeneity: Chi² = 20.40, df = 18 (P = 0.31); I² = 12% | | | | | | |
| Test for overall effect: Z = 5.65 (P < 0.00001) | | | | | | |
| Test for subgroup differences: Chi² = 0.50, df = 1 (P = 0.48), I² = 0% | | | | | | |

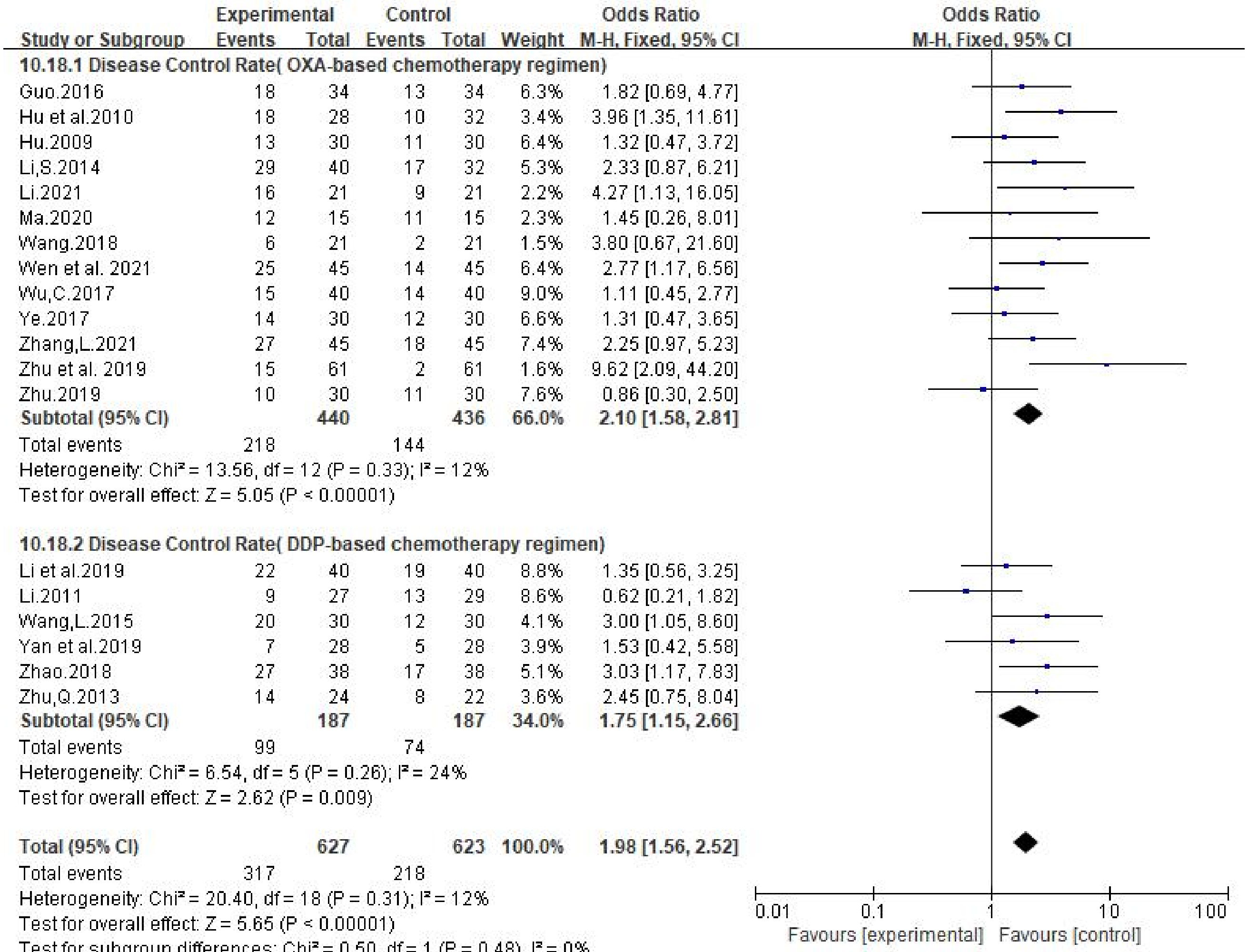


**Figure S13. DCR Subgroup Analysis of Chemotherapy Regimens Using platinum**

| Study or Subgroup | Experimental Events | Experimental Total | Control Events | Control Total | Weight | Odds Ratio M-H, Fixed, 95% CI |
|---|---|---|---|---|---|---|
| **10.10.1 Disease Control Rate( <8 weeks)** | | | | | | |
| Li et al.2019 | 22 | 40 | 19 | 40 | 6.9% | 1.35 [0.56, 3.25] |
| Li,S.2014 | 29 | 40 | 17 | 32 | 4.2% | 2.33 [0.87, 6.21] |
| Wen et al. 2021 | 25 | 45 | 14 | 45 | 5.0% | 2.77 [1.17, 6.56] |
| Yan et al.2019 | 7 | 28 | 5 | 28 | 3.0% | 1.53 [0.42, 5.58] |
| Zhang et al.2005 | 14 | 30 | 12 | 30 | 5.2% | 1.31 [0.47, 3.65] |
| Zhu,Q.2013 | 14 | 24 | 8 | 22 | 2.8% | 2.45 [0.75, 8.04] |
| **Subtotal (95% CI)** | | **207** | | **197** | **27.2%** | **1.89 [1.26, 2.84]** |
| Total events | 111 | | 75 | | | |
| Heterogeneity: Chi² = 2.25, df = 5 (P = 0.81); I² = 0% | | | | | | |
| Test for overall effect: Z = 3.06 (P = 0.002) | | | | | | |
| **10.10.2 Disease Control Rate( ≥8 weeks)** | | | | | | |
| Guo.2016 | 18 | 34 | 13 | 34 | 5.0% | 1.82 [0.69, 4.77] |
| Hu et al.2010 | 18 | 28 | 10 | 32 | 2.7% | 3.96 [1.35, 11.61] |
| Hu.2009 | 13 | 30 | 11 | 30 | 5.0% | 1.32 [0.47, 3.72] |
| Li,S.2014 | 29 | 40 | 17 | 32 | 4.2% | 2.33 [0.87, 6.21] |
| Li.2011 | 9 | 27 | 13 | 29 | 6.8% | 0.62 [0.21, 1.82] |
| Li.2021 | 16 | 21 | 9 | 21 | 1.7% | 4.27 [1.13, 16.05] |
| Ma.2020 | 12 | 15 | 11 | 15 | 1.8% | 1.45 [0.26, 8.01] |
| Sun et al. 2013 | 11 | 20 | 7 | 20 | 2.5% | 2.27 [0.64, 8.11] |
| Wei.2018 | 6 | 21 | 2 | 21 | 1.2% | 3.80 [0.67, 21.60] |
| Wen et al. 2021 | 25 | 45 | 14 | 45 | 5.0% | 2.77 [1.17, 6.56] |
| Wu,C.2017 | 15 | 40 | 14 | 40 | 7.1% | 1.11 [0.45, 2.77] |
| Ye.2017 | 14 | 30 | 12 | 30 | 5.2% | 1.31 [0.47, 3.65] |
| Zhang,L.2021 | 27 | 45 | 18 | 45 | 5.8% | 2.25 [0.97, 5.23] |
| Zhang.2017 | 20 | 40 | 12 | 40 | 4.9% | 2.33 [0.93, 5.84] |
| Zhao.2018 | 27 | 38 | 17 | 38 | 4.0% | 3.03 [1.17, 7.83] |
| Zhu et al. 2019 | 15 | 61 | 2 | 61 | 1.2% | 9.62 [2.09, 44.20] |
| Zhu,Q.2013 | 14 | 24 | 8 | 22 | 2.8% | 2.45 [0.75, 8.04] |
| Zhu.2019 | 10 | 30 | 11 | 30 | 5.9% | 0.86 [0.30, 2.50] |
| **Subtotal (95% CI)** | | **589** | | **585** | **72.8%** | **2.05 [1.61, 2.62]** |
| Total events | 299 | | 201 | | | |
| Heterogeneity: Chi² = 19.10, df = 17 (P = 0.32); I² = 11% | | | | | | |
| Test for overall effect: Z = 5.74 (P < 0.00001) | | | | | | |
| **Total (95% CI)** | | **796** | | **782** | **100.0%** | **2.01 [1.63, 2.48]** |
| Total events | 410 | | 276 | | | |
| Heterogeneity: Chi² = 21.41, df = 23 (P = 0.56); I² = 0% | | | | | | |
| Test for overall effect: Z = 6.50 (P < 0.00001) | | | | | | |
| Test for subgroup differences: Chi² = 0.11, df = 1 (P = 0.74), I² = 0% | | | | | | |

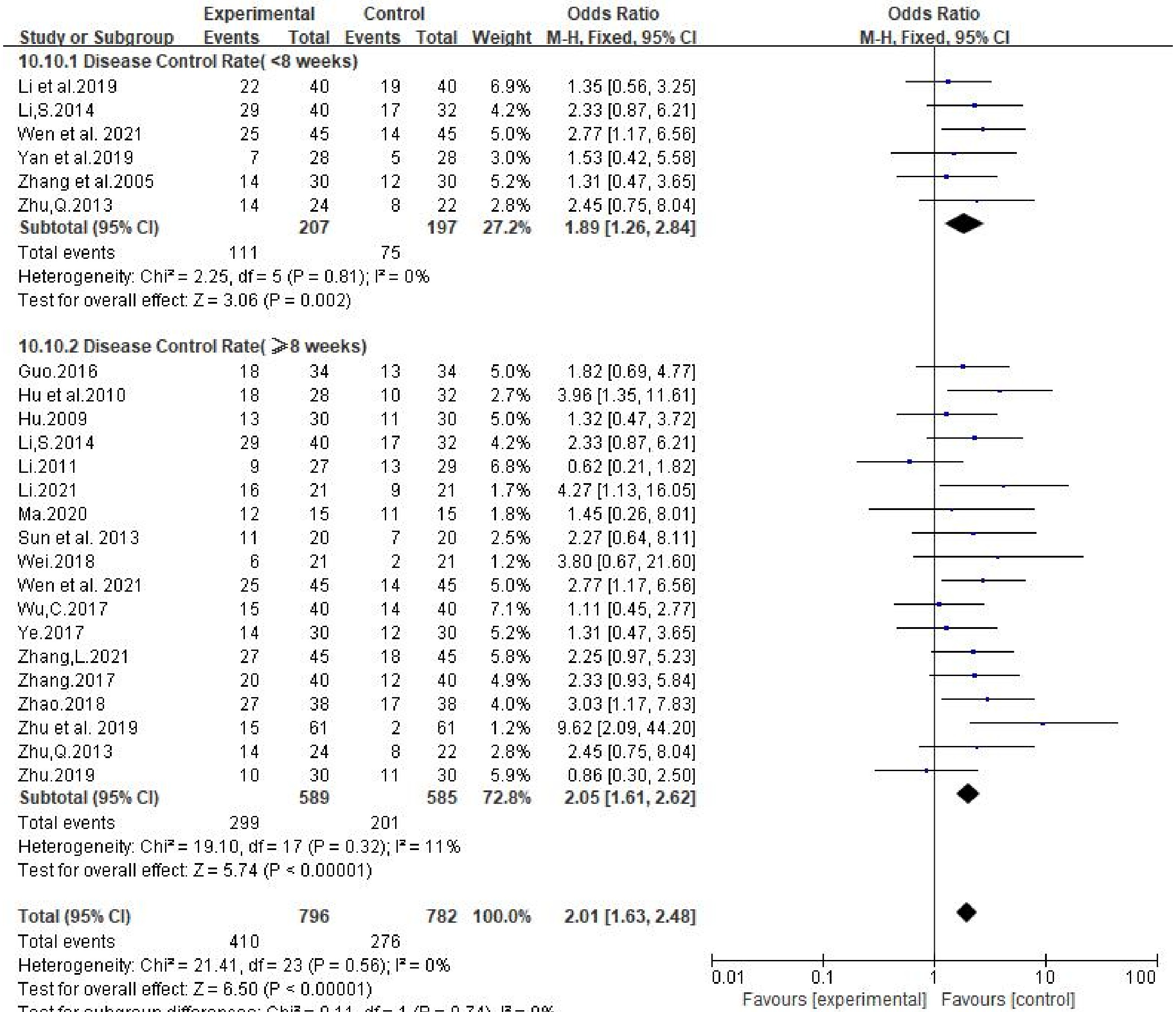


**Figure S14. Subgroup analysis of DCR via treatment duration**
